# Di- and Tri-nuclear V^III^ and Cr^III^ Complexes of Dipyridyltriazoles: Ligand Rearrangements, Mixed Valency and Ferromagnetic Coupling

**DOI:** 10.3389/fchem.2020.00540

**Published:** 2020-07-09

**Authors:** Julia Rinck, Jonathan A. Kitchen, Anthony B. Carter, Yanhua Lan, Christopher E. Anson, Karin Fink, Sally Brooker, Annie K. Powell

**Affiliations:** ^1^Institute of Inorganic Chemistry, Karlsruhe Institute of Technology, Karlsruhe, Germany; ^2^Department of Chemistry and MacDiarmid Institute for Advanced Materials and Nanotechnology, University of Otago, Dunedin, New Zealand; ^3^Chemistry, School of Natural and Computational Sciences, Massey University, Auckland, New Zealand; ^4^Institute of Nanotechnology, Karlsruhe Institute of Technology, Eggenstein-Leopoldshafen, Germany

**Keywords:** vanadium, chromium, triazole, trinuclear, deamination, rearrangement, ferromagnetic coupling, helicate

## Abstract

The first dinuclear and trinuclear chromium(III) and dinuclear vanadium(III) complexes of *N*^4^-**R**-substituted-3,5-di(2-pyridyl)-1,2,4-triazole (**Rdpt**) ligands have been prepared by solvothermal complexations under inert atmospheres, and characterized. The reactions of Cr^III^ and V^III^ with **adpt** (**R** = amino) resulted in deamination of the ligand and yielded the dinuclear doubly-triazolate bridged complexes [${\text{V}}_{2}^{\text{III}}$(**dpt**^−^)_2_Cl_4_] (**1**) and [${\text{Cr}}_{2}^{\text{III}}$(**dpt**^−^)_2_Cl_4_] (**2**). In the case of the Cr^III^ complex **2** this bridging results in a rare example of ferromagnetic coupling for a dinuclear Cr^III^ compound. DFT studies confirm that in **2** the ferromagnetic coupling pathways dominate over the antiferromagnetic pathways, whereas in **1** the reverse occurs, consistent with the observed overall antiferromagnetic coupling in that case. It was also found that the use of different additives in the reaction allows the nuclearity of the Cr^III^ product to be manipulated, giving either the dinuclear system, or the first example of a trinuclear circular helicate for a **Rdpt** complex, [${\text{Cr}}_{3}^{\text{III}}$(**dpt**)_3_Cl_6_]·1¾MeCN·¼DCM (**3**). Reaction of ***N***^**4**^***-*pydpt (R** = 4-pyridyl) with V^III^ led to an unusual shift of the pyridyl substituent from *N*^4^ to *N*^1^ of the triazole, forming the ligand isomer ***N***^**1**^-**pydpt**, and giving a dinuclear doubly-triazole bridged complex, [${\text{V}}_{2}^{\text{III}}$(***N***^**1**^-**pydpt**)_2_Cl_6_]·2MeCN (**4**). Reaction with Cr^III^ results in loss of the 4-pyridyl ring and a mixture of the di- and trinuclear complexes, **2** and **3**. Interestingly, partial oxidation of the V^III^ in dinuclear complex **4** to vanadyl V^IV^=O was identified by crystallographic analysis of partially oxidized single crystals, [(V^IV^O)_0.84_(V^III^)_1.16_(***N***^**1**^-**pydpt**)_2_Cl_5.16_]·0.84H_2_O·1.16MeCN (**5**).

## Introduction

**Figure 1:**
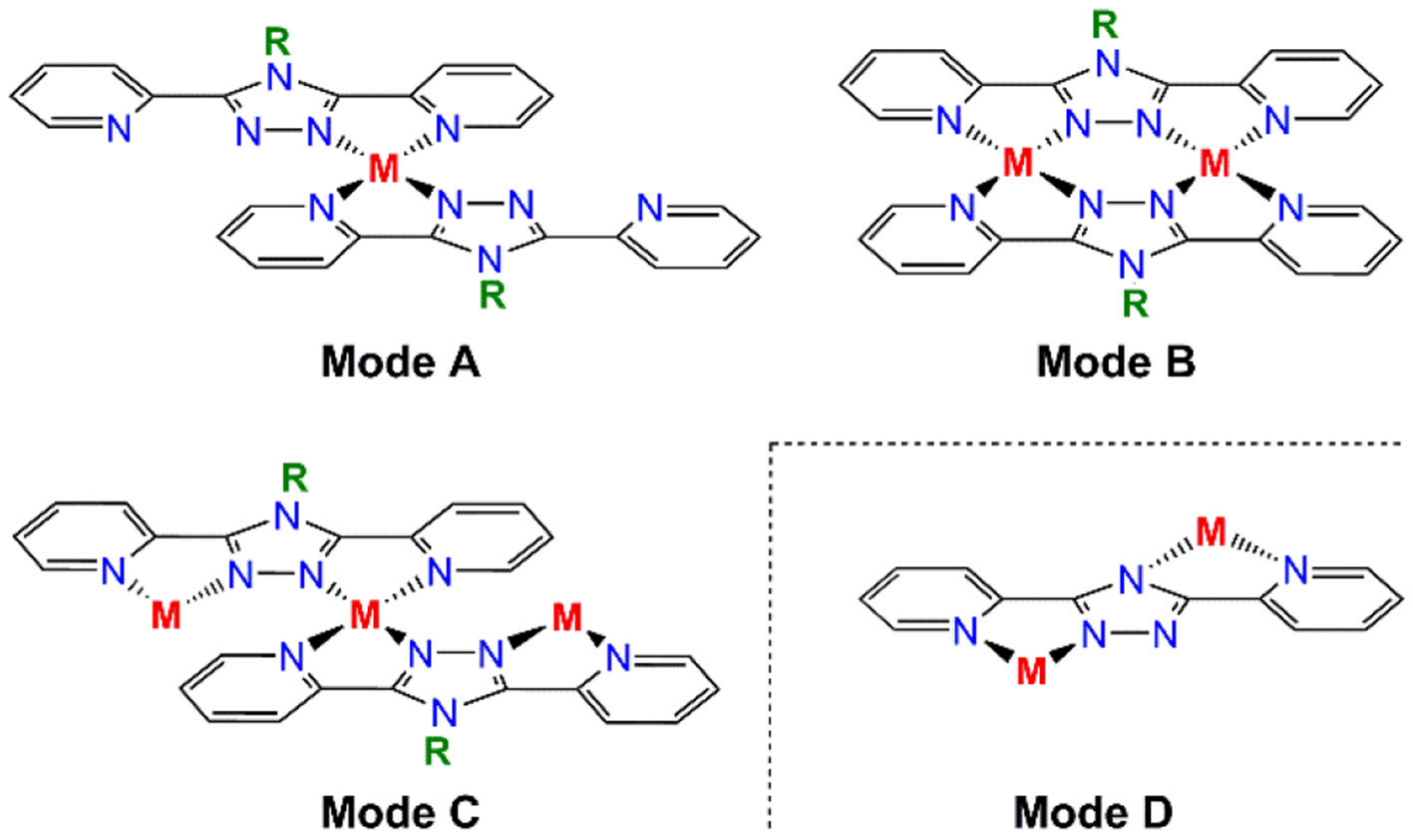
Some of the binding modes observed for R-substituted-3,5-dipyridyl-4H-1,2,4-triazole type ligands (Rdpt), including a binding mode only available to the triazolate dpt^−^ analogs in which R is absent (mode D).

Transition metal complexes of 1,2,4-triazoles have attracted significant attention over the last two decades (Kunkeler et al., 1996; Haasnoot, 2000; Klingele and Brooker, 2003; Klingele et al., 2005; Kitchen and Brooker, 2008; Aromí et al., 2011; Miller and Brooker, 2016; Bushuev et al., 2017; Chen et al., 2017, 2018; Feltham et al., 2017; Murphy et al., 2017; Rodríguez-Jiménez et al., 2017; Clements et al., 2018; Hogue et al., 2018; Li et al., 2018; Liu et al., 2018; Zenere et al., 2018). Much of this interest has stemmed from the spin crossover (SCO) phenomenon that is frequently observed in octahedral iron(II) complexes of 1,2,4-triazoles (Kunkeler et al., 1996; Haasnoot, 2000; Klingele and Brooker, 2003; Klingele et al., 2005; Kitchen and Brooker, 2008; Miller and Brooker, 2016; Bushuev et al., 2017; Chen et al., 2017, 2018; Feltham et al., 2017; Murphy et al., 2017; Rodríguez-Jiménez et al., 2017; Clements et al., 2018; Hogue et al., 2018; Li et al., 2018; Liu et al., 2018; Zenere et al., 2018). Additionally, exchange coupling between triazole bridged metal centers has resulted in families of dinuclear, trinuclear and even polymeric complexes being targeted for the development of magnetically interesting complexes (Hogue et al., 2018). The 1,2,4-triazole moiety can be introduced into a variety of different ligand scaffolds, however a particularly versatile and attractive class of ligand is that of the *N*^4^-**R-**substituted-3,5-di(2-pyridyl)-1,2,4-triazole (**Rdpt**) family (Klingele and Brooker, 2003; Kitchen and Brooker, 2008; Feltham et al., 2017). **Rdpt** ligands are ideal candidates in terms of our interest in developing multi-nuclear coordination complexes as they offer a rich variety of coordination and bridging modes (Figure 1). Indeed these ligands have led to the formation of well over a hundred complexes to date, featuring a wide range of nuclearities (mono-, di-, tri-, tetra-, and octa-nuclear through to polymeric), metal to ligand ratios (including 1:1, 1:2, 1:3, 2:1 and 2:2 and 3:4) and **Rdpt**/**dpt**^**−**^ binding modes (Figure 1).

So far, work with **Rdpt** ligands has primarily focused on systems incorporating the mid to late 3*d* transition metal ions. To the best of our knowledge no **Rdpt** or 3,5-di(2-pyridyl)-1,2,4-triazolate (**dpt**^**−**^) complexes of the earlier transition metal ions Cr^III^ and V^III^ have been reported. Thus, the work presented herein builds on our previous investigations of **Rdpt**-based systems but now targets the first examples of complexes of V^III^ and Cr^III^. Of particular interest is the formation of multi-nuclear Cr^III^ complexes as the ferromagnetic coupling between Cr^III^ centers leads to magnetically interesting compounds with large numbers of unpaired spins (Tono et al., 2003; Rinck et al., 2010; Døssing, 2014). Ferromagnetic coupling can be achieved by careful consideration of the bridging species used to connect the metal centers, and given the proclivity of 1,2,4-triazoles to act as bridging moieties, **Rdpt**-based systems are ideal candidates to facilitate this approach.

Of the wide range of **Rdpt** ligands available, 4-amino-3,5-di-2pyridyl-4H-1,2,4-triazole (**adpt**) has been the most extensively studied to date with over 80 metal complexes structurally characterized. The literature complexes of **adpt** are almost solely mono- or dinuclear (Klingele and Brooker, 2003), with the exception of some tetranuclear (White and Brooker, 2013), octanuclear (White and Brooker, 2013), and polymeric (Dupouy et al., 2009) systems. The next most commonly studied ligand of this general type is **dpt**^**−**^ with the resulting complexes showing a far richer variety in nuclearity from monometallic through to polymetallic clusters and polymers (Zhang et al., 2005; Chen et al., 2006a; Keene et al., 2006; White et al., 2012).

**Figure 2:**
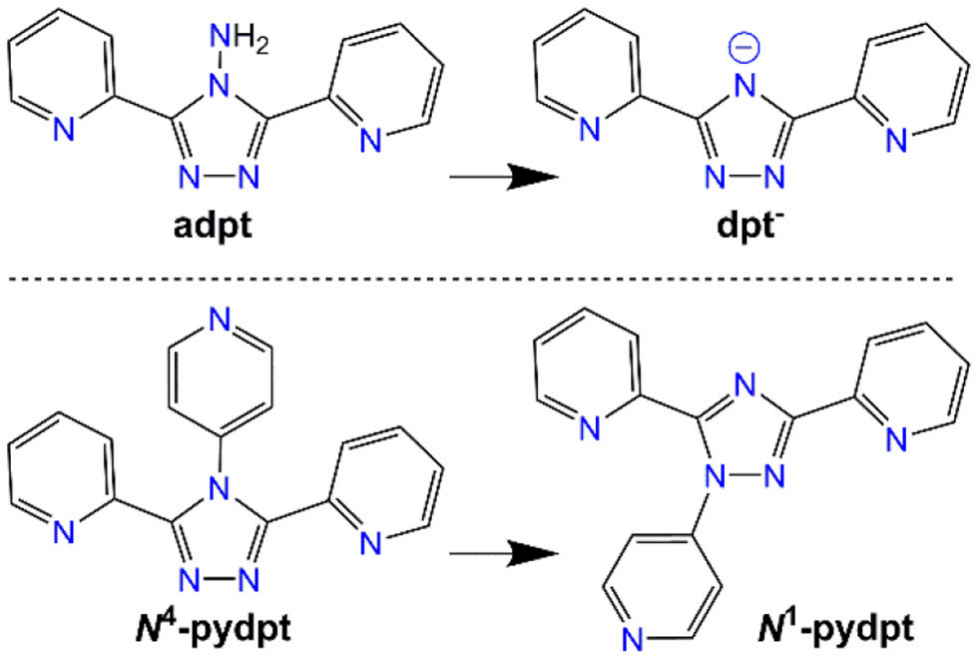
The two Rdpt ligands used in this study, adpt (R = NH_2_) and *N*^4^-pydpt (R = 4-pyridyl), and the respective rearrangements (deamination; (White et al., 2012) and *N*^4^- to *N*^1^-pyridyl shift; (Kitchen et al., 2010b); see text for more detail), they undergo during complexation, resulting in complexes of dpt^−^ and *N*^1^-pydpt.

Herein we detail the synthesis, structures and magnetic behavior of the first ever examples of vanadium and chromium complexes of such **Rdpt** ligands. Specifically we present here: [${\text{V}}_{2}^{\text{III}}$(**dpt**)_2_Cl_4_] (**1**), [${\text{Cr}}_{2}^{\text{III}}$(**dpt**)_2_Cl_4_] (**2**) and [${\text{Cr}}_{3}^{\text{III}}$(**dpt**)_3_Cl_6_]·1¾MeCN·¼DCM (**3**), as well as two products of complexation of 4-pyridyl-3,5-di-2pyridyl-4H-1,2,4-triazole **(*N***^**4**^-**pydpt**, Figure 2), [${\text{V}}_{2}^{\text{III}}$(***N***^**1**^-**pydpt**)_2_Cl_6_]·2MeCN (**4**) and its air oxidation product [(V^IV^O)_0.84_(V^III^)_1.16_(***N***^**1**^-**pydpt**)_2_Cl_5.16_]·0.84H_2_O· 1.16MeCN (**5**).

## Results and Discussion

### Complexation of Adpt With Cr^III^ and V^III^

A 2:1 mixture of **adpt** and VCl_3_ in acetonitrile and dichloromethane was sealed in a Teflon lined autoclave under argon and heated at 150°C for 45 h. Slow cooling to room temperature gave orange needle crystals of a dinuclear complex of the deaminated ligand **dpt**^**−**^ (Figure 2), [V^III^(**dpt**)Cl_2_]_2_ (**1**).

The analogous chromium complex, [Cr^III^(**dpt**)Cl_2_]_2_ (**2**), can be obtained in the form of dichroic red-green crystals by replacing VCl_3_ by ${\text{Cr}}_{2}^{\text{II}}$(OAc)_4_(H_2_O)_2_ and adding either pivalic acid or cobalt pivalate, Co_2_(H_2_O)(piv)_4_(Hpiv)_4_. Interestingly, one of these two additives must be present in order to obtain **2**. When the reaction was repeated with **adpt** and CrCl_2_, the same [Cr^III^(**dpt**)Cl_2_]_2_ (**2**) product was obtained but with a side product of a gray precipitate that could not be easily separated from **2**. When the complexation was instead carried out on the deaminated ligand, H**dpt**, the crystal quality of **2** was significantly poorer than that obtained from the original protocol.

With the addition of pivalic acid or cobalt pivalate to the solvothermal reaction seemingly important for the successful and clean formation of **2**, other additives [e.g., GdCl_3_, diaminomaleonitrile, NaN_3_, KSCN, and Co(OAc)_2_·4H_2_O] were tested in an attempt to access different structural variants. Interestingly, the only attempt that resulted in a crystalline material was when **adpt** and ${\text{Cr}}_{2}^{\text{II}}$(OAc)_4_(H_2_O)_2_ were reacted in a 1.5:1 ratio with an additional 3 equivalents of GdCl_3_. In this instance, brown irregular single crystals of a *triangular* trinuclear complex [Cr_3_(**dpt**)_3_Cl_6_]·1¾MeCN·¼DCM (**3**) were obtained.

The deamination of **adpt** during complexation has been observed previously in the reaction of Cu^II^-acetate with **adpt**, resulting in the octanuclear grid complex, [${\text{Cu}}_{8}^{\text{II}}$(**dpt**)_4_(OH)_4_(OAc)_8_], (White et al., 2012) which is the largest example of a discrete **dpt**^**−**^ complex. In this particular case the N-N bond cleavage was proposed to be induced by the transition metal ion, and that this turn might well be an important factor in the determining the structure of the product. The analogous reaction with H**dpt** instead yielded a tetranuclear complex, [${\text{Cu}}_{4}^{\text{II}}$(**dpt**)_2_(OAc)_4_(OMe)(OH)]. In the present case, not only are transition metal ions present, but also the reactions are carried out under harsher reaction conditions.

### Complexation of *N*^4^-pydpt With Cr^III^ and V^III^

**Figure 3:**
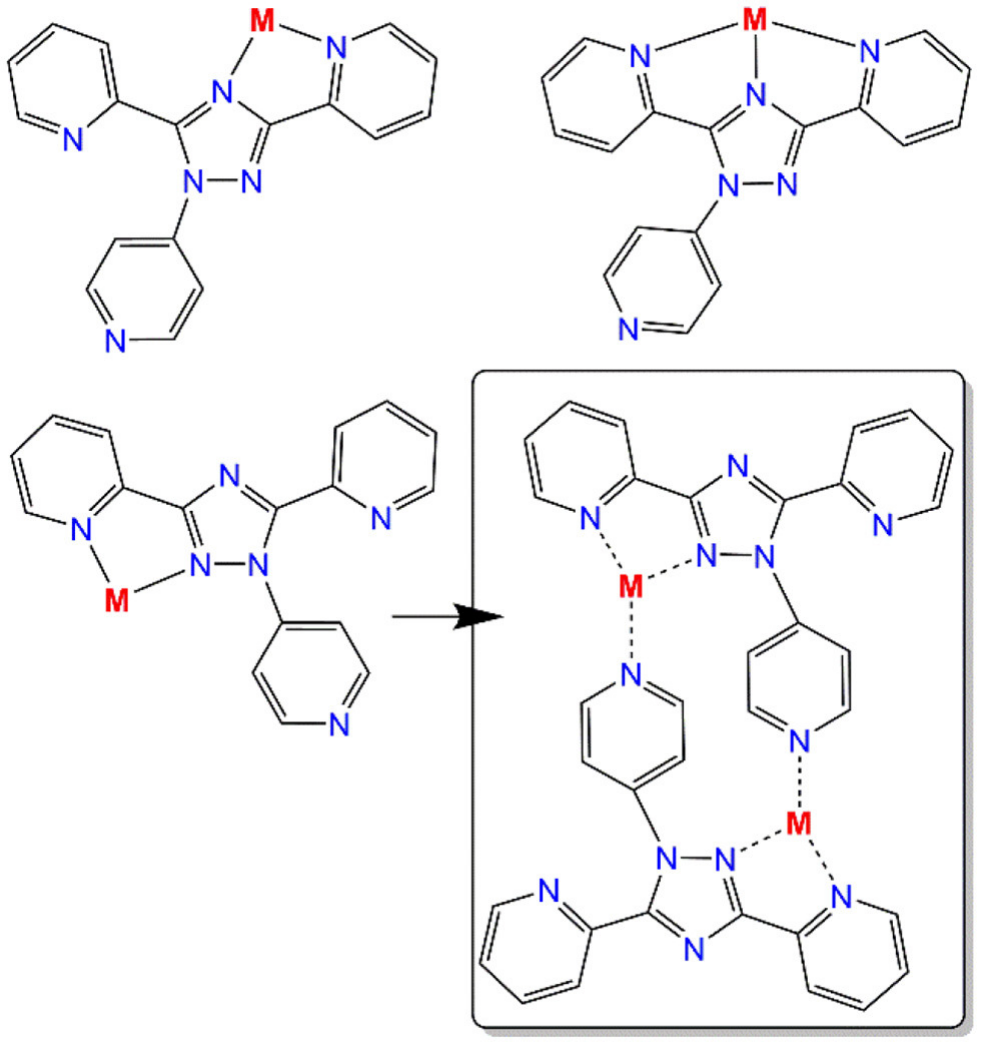
Possible bi- and tri-dentate binding pockets of the rearranged *N*^1^-pyridyl-3,5-di(2-pyridyl)-1,2,4-triazole ligand (*N*^1^-pydpt) and the observed binding mode in dinuclear complex 4 (box) which also features bridging by the 4-pyridyl substituents at *N*^1^ of the triazole rings.

Altering the *N*^4^-substituent in **Rdpt** systems allows the properties of the resulting complexes to be significantly altered (Feltham et al., 2017; Hogue et al., 2018). By including an additional coordination site, such as a 4-pyridyl ring as in the present case (***N***^**4**^-**pydpt**, Figure 2), it is possible to generate extended networks of metal complexes or systems where another metal ion bridges two complexes. Interestingly, during previous complexations of ***N***^**4**^-**pydpt**, a rearrangement of the pyridyl group from N^4^ to N^1^ of the triazole ring has been observed (Figure 2), with the N^4^ substituted compound, 4-pyridyl-3,5-di-2pyridyl-4H-1,2,4-triazole (***N***^**4**^-**pydpt**) being the kinetic and *N*^1^ (***N***^**1**^-**pydpt**) the thermodynamic products (Kitchen et al., 2010b). Whilst ***N***^**4**^-**pydpt** has the same variety of binding modes as other **Rdpt** systems, for the rearranged ***N***^**1**^**-pydpt** system a range of alternative binding modes can be anticipated (Figure 3)—whilst the *N*^1^*N*^2^-triazole bridging mode is precluded.

The reaction of ***N***^**4**^-**pydpt** and VCl_3_ in 1:1.3 ratio, again in acetonitrile and dichloromethane and sealed in a Teflon lined autoclave under argon and heated at 150°C for 50 h, then slowly cooled to room temperature, resulted in green block-like crystals of the dinuclear complex of the rearranged ligand, [${\text{V}}_{2}^{\text{III}}$(***N***^**1**^**-pydpt**)_2_Cl_6_]·2MeCN (**4**) (see below). Storage of crystals of the dinuclear V^III^ complex **4** under ambient conditions led to partial oxidation, giving [(V^IV^O)_0.84_(V^III^)_1.16_(***N***^**1**^-**pydpt**)_2_Cl_5.16_]·0.84H_2_O·1.16MeCN (**5**) which has also been structurally characterized (see below). All attempts to reproduce (**4**) were unsuccessful and yielded a complex with partial oxidation in all cases. These reactions included using previously unopened VCl_3_ from two different commercial suppliers, freshly synthesized ***N***^**4**^-**pydpt** as well as different gloveboxes and autoclaves.

Interestingly, the analogous reaction using 1 equivalent of CrCl_2_ instead of 1.3 equivalents of VCl_3_ does not result in the isostructural complex, but rather a mixture of two chromium complexes: the triangular trinuclear complex [Cr_3_(**dpt**)_3_Cl_6_]·1¾MeCN·¼DCM (**3**) and the dinuclear complex **2** where the 4-pyridyl group has been completely cleaved from the triazole ring to give **dpt**^**−**^.

### Crystal Structures of 1–5

**Figure 4:**
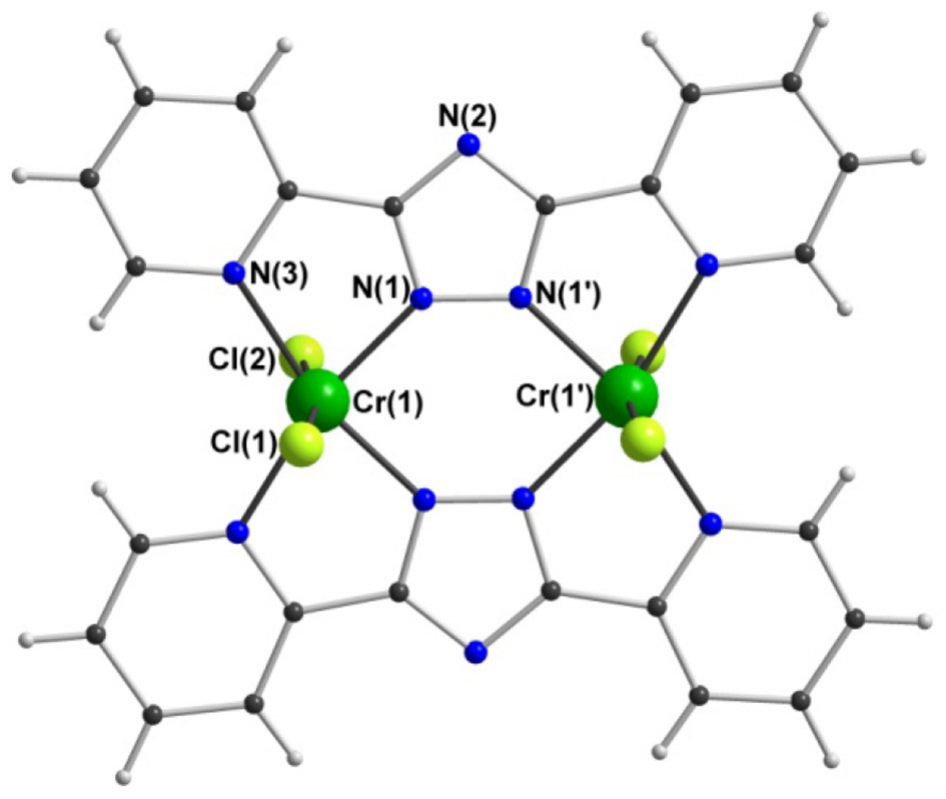
Molecular structure of [${\text{Cr}}_{2}^{\text{III}}$(dpt)_2_Cl_4_] (2). [${\text{V}}_{2}^{\text{III}}$(dpt)_2_Cl_4_] (1) is isostructural.

The two isostructural dinuclear compounds **1** and **2** (Figure 4, Table 1) crystallize in the orthorhombic space group *Pnnm* with two formula units per unit cell. The complex molecule occupies a site of 2/m symmetry in the crystal, with the Cr and Cl atoms lying in the mirror plane, the twofold axis passing through N(2) and N(2'), and an inversion center midway between the two Cr centers. The asymmetric unit thus contains a quarter of the molecule.

**Table 1:** Selected bond distances (Å) and angles (°) for compounds 1–5.

	**1**	**2**	**3**	**4**	**5^a^**
Metal	${\text{V}}_{2}^{\text{III}}$	${\text{Cr}}_{2}^{\text{III}}$	${\text{Cr}}_{3}^{\text{III}}$	${\text{V}}_{2}^{\text{III}}$	${\text{V}}_{1.16}^{\text{III}}$${\text{V}}_{0.84}^{\text{IV}}$ ^a^
M-N(trz)	2.044 (2)	2.008 (2)	2.022 (3)−2.103 (3)	2.197 (3)	2.270 (2)
M-N(pyr)	2.184 (3)	2.132 (2)	2.071 (3)−2.125 (3)	2.166 (3) N3	2.161 (2) N3
				2.181 (3) N4	2.168 (2) N4
M-Cl(1)	2.3150 (13)	2.3049 (11)	2.2813 (10)−2.2955 (11)	2.333 (2)	2.361 (9)
M-Cl(2)	2.3136 (13)	2.3080 (12)	2.2838 (11)−2.2935 (11)	2.315 (2)	2.324 (9)
M-X(3)^a^				Cl (3)^a^ 2.272 (2)	O(3b)^a^ 1.774 (8)
M ··· M	4.2570 (14)	4.2144 (12)	6.0428 (8)−6.0988 (8)	7.487 (2)	7.5559 (11)
*cis* D-M-D^b^	75.51 (9)−119.22 (14)	77.28 (9)−116.34 (14)	77.01 (12)−103.20 (12)	76.11 (9)−100.32 (9)	75.01 (9)−100.87 (7)
*trans* D-M-D^b^	160.96 (5)−165.27 (10)	166.38 (9)−168.83 (4)	160.14 (12)−173.02 (9)	168.77 (7)−175.51 (9)	166.6 (3)−172.43 (9)
N(pyr)-M-N(trz)^c^	75.51 (9)	77.28 (9)	77.01 (12)−79.62 (12)	76.11 (9)	75.01 (9)
N(pyr)-M-N(trz)^d^	165.27 (10)	166.38 (9)	83.31 (12)−96.45 (12)	100.32 (9)	98.28 (9)
Cl-M-Cl	160.96 (5)	168.84 (5)	93.71 (4)−95.27 (4)	94.03 (4)−169.12 (3)	92.20 (7)−166.90 (4)
Σ	104.6	80.4	62.5-74.8	55.4	63.5, 63.1^a^

a * Partial replacement of the equatorial chloride ligand Cl(3) in 4 by a vanadyl oxygen atom O(3b) in 5. The relative occupancies of Cl(3a):O(3b) in 5 were refined with the V1-Cl(3a) distance restrained (DFIX) to the value for V1-Cl3 found in the fresh crystals (2.272 Å) and rigid-bond restraints (RIGU) were applied to the thermal parameters of V(1), Cl(3A), and O(3B); the occupancies converged at 58:42*.

b * D is any donor atom coordinated to M*.

c * N atoms from same ligand*.

d * N atoms from different ligands*.

The two anionic bis-bidentate **dpt**^**−**^ ligands provide a planar equatorial N_4_-donor set (two pyridyl and two triazole nitrogen atoms) to each metal(III) center, and doubly bridge them in a *N'N*^1^*N*^2^*N”*-bridging mode (Mode B in Figure 1). The N_4_Cl_2_ geometry can be described as distorted octahedral with Σ values of 104.6° for V^III^ **1** and 80.4° for Cr^III^ **2**. Here Σ is the sum of the absolute values of the difference between the 12 cis angles and 90° and a value of 0 would indicate a perfect octahedron. The coordinated chloride ions occupy the axial positions. Consistent with previous complexes, the M-N_pyridyl_ bond lengths are longer than the M-N_triazole_ bond lengths and the Cl-M-Cl angle is ≠ 180° (Table 1). The M···M separation is 4.209(2) Å in [Cr_2_(**dpt**)_2_Cl_4_] and 4.257(2) Å in [V_2_(**dpt**)_2_Cl_4_] which is again consistent with other dinuclear 3d complexes of **dpt**^−^.

**Figure 5:**
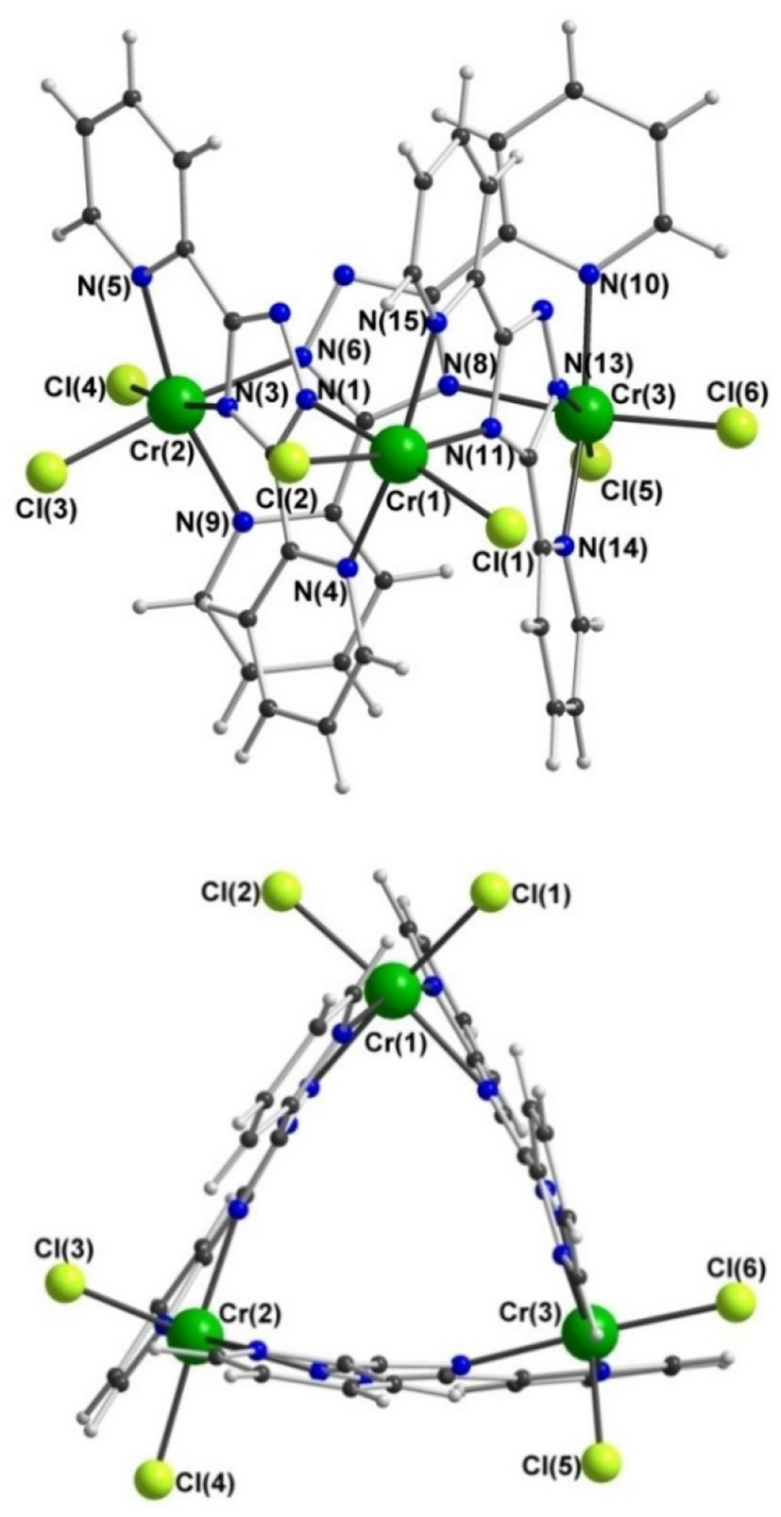
Two views of the molecular structure of [Cr^III^(dpt)Cl_2_]_3_·1.75MeCN·0.25CH_2_Cl_2_ (3); for clarity solvents of crystallization are not shown.

In contrast, trinuclear compound **3** crystallizes in the monoclinic space group *P*2_1_/*c* with four formula units per unit cell, so the entire complex is in the asymmetric unit (Figure 5, Table 1). The triangular configuration of [Cr_3_(**dpt**)_3_Cl_6_] is interesting, as to the best of our knowledge the only trinuclear complexes of **Rdpt** ligands reported before are a linear trinuclear complex (Figure 1, mode C) (Kitchen et al., 2010b) and a 1D-ladder of chloride bridged trinuclear units (Chen et al., 2006b). This makes **3** the first example of a molecular triangle in this family and, indeed, the first trinuclear helicate (Albrecht, 2001; Zangrando et al., 2009; Lippert and Sanz Miguel, 2011; Thomas, 2011; Hogue et al., 2017; Zhang et al., 2018) to feature an **Rdpt** ligand. The three Cr^III^ centers are crystallographically independent but in all cases are coordinated by two bidentate **dpt**^**−**^ ligands, one bound by *N'N*^1^ and the other by *N”N*^4^ (Figure 1, mode D), which also bridge the adjacent Cr^III^ centers in the triangle. Two chloride ions, this time bound in a *cis* configuration [Cl-Cr-Cl range=93.7-95.3°], complete the octahedral coordination sphere of each Cr^III^ center, giving a neutral complex overall. The resulting N_4_Cl_2_ coordination sphere is again best described as distorted octahedral, with Σ values of 63.0, 62.5, and 74.8° which are lower values than those observed in the dimeric structure of **2 (**80.4°). The bridging mode adopted by the **dpt**^**−**^ ligands is significantly different to the *N*^1^ and *N*^2^ triazolate nitrogen atom bridging seen in **2** (Figure 1, mode B), as in **3** each **dpt**^**−**^ ligand bridges two Cr^III^ atoms through the *N*^1^ and *N*^4^ triazolate nitrogen atoms (*N'N*^1^*N*^4^*N”*-bridging mode, Figure 1, mode D), a mode which is commonly observed in multi-nuclear **dpt**^**−**^ based complexes (Klingele and Brooker, 2003; Kitchen and Brooker, 2008; Feltham et al., 2017). Unsurprisingly, this results in a much greater Cr···Cr separation in **3** [6.0428(8)−6.0989(8) Å] than in **2** [4.209(2) Å], consistent with other complexes that exhibit this bridging mode. Unfortunately, it was not possible to obtain a pure “bulk” sample of these crystals so magnetic analysis was not possible.

**Figure 6:**
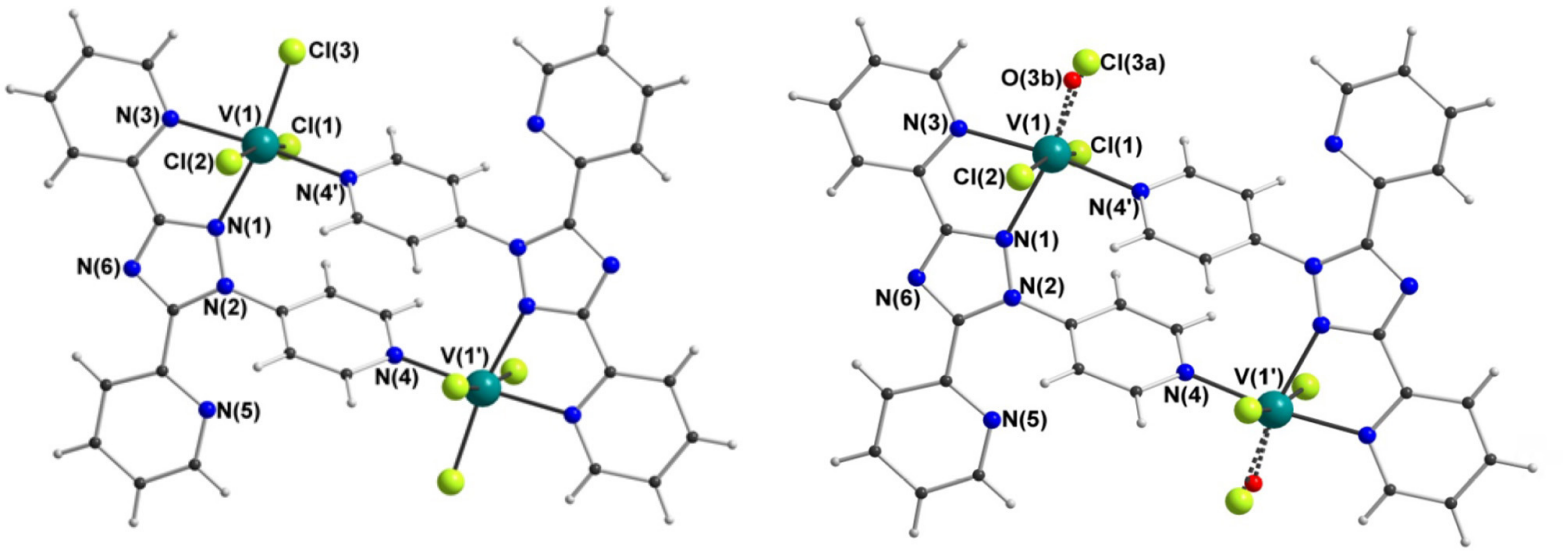
Molecular structure of [V^III^(*N*^1^-pydpt)Cl_3_]_2_·2MeCN (4) (left) and [(V^IV^O)_0.84_(V^III^)_1.16_(*N*^1^-pydpt)_2_Cl_5.16_]·0.84H_2_O·1.16MeCN (5) (right); for clarity the solvents of crystallization are not shown.

X-ray crystallography carried out on a fresh sample of the green crystals obtained from the reaction between ***N***4**-pydpt** and VCl_3_, dinuclear [V^III^(***N***^**1**^**-pydpt**)Cl_3_]_2_·2MeCN (**4**), reveal that it crystallizes in the triclinic space group *P*-1 with one formula unit per unit cell. Hence the asymmetric unit comprises half of the complex with the other half generated by a center of inversion (Figure 6, Table 1). The two V^III^ centers are doubly bridged by two rearranged ligand molecules meaning that during the solvothermal complexation reaction the *N*^4^ pyridyl substituent has migrated to the *N*^1^ position. As noted above, this behavior has been observed previously. Such rearrangements are often thermally induced (Jørgensen et al., 2001; Kitchen et al., 2010a). The V^III^ center is coordinated in a bidentate pocket formed by the *N*^2^ atom of the triazole and the nitrogen atom of the 2-pyridyl moiety of the ***N***^**1**^**-pydpt** as well by the nitrogen atom of the 4-pyridyl moiety of a second ***N***^**1**^**-pydpt** ligand. The 4-pyridyl rings are rotated out of the plane of the attached 3,5-*bis*-(2-pyridyl)-4H-1,2,4-triazole ligand moiety with a torsion angle of 63.5°. The N_3_Cl_3_ distorted octahedral (Σ = 55.4°) coordination sphere is completed by three chloride ions arranged in a *mer* configuration. The observed bridging mode adopted here results in a large V···V separation of 7.487(2) Å suggesting that little or no magnetic exchange coupling is expected, even given the conjugation present. Unfortunately, it has not proved possible to collect data on the purely V^III^ compound, **4** as explained above.

Single crystals of the dinuclear V^III^ complex **4** were re-investigated after storage under ambient conditions, revealing a partial oxidation had taken place to give [(V^IV^O)_0.84_(V^III^)_1.16_(***N***^**1**^-**pydpt**)_2_Cl_5.16_]·0.84H_2_O·1.16MeCN (**5**) (Figure 6, Table 1). The molecular structure of the fresh material, **4**, is essentially maintained, but the two symmetry-equivalent vanadium centers have now been partially oxidized to V^IV^. Refinement of the structure of **5**, including modeling the corresponding partial replacement of the equatorial chloride ligand, Cl3, by a vanadyl oxo ligand, O3b [V1-O3b = 1.774(8) vs. V1–Cl3 = 2.333(2) Å], to balance the charge associated with the partial oxidation from V^III^ to V^IV^, as well as the modeling of the partial replacement of the lattice acetonitrile molecules by water molecules, is consistent with the aged crystal of **5** comprising a 58:42 ratio of V^III^: V^IV^ [and Cl3:O3b and MeCN:H_2_O]. Comparing the V-donor bond lengths of partially oxidized **5** with those of the non-oxidized V^III^ compound **4**, the V1-N1 distance is significantly elongated, which is to be expected as it is *trans* to the vanadyl oxygen, while the two axial V-Cl bond lengths show a smaller increase (Table 1).

### Magnetic Studies of 1, 2, 5, and 6

**Figure 7:**
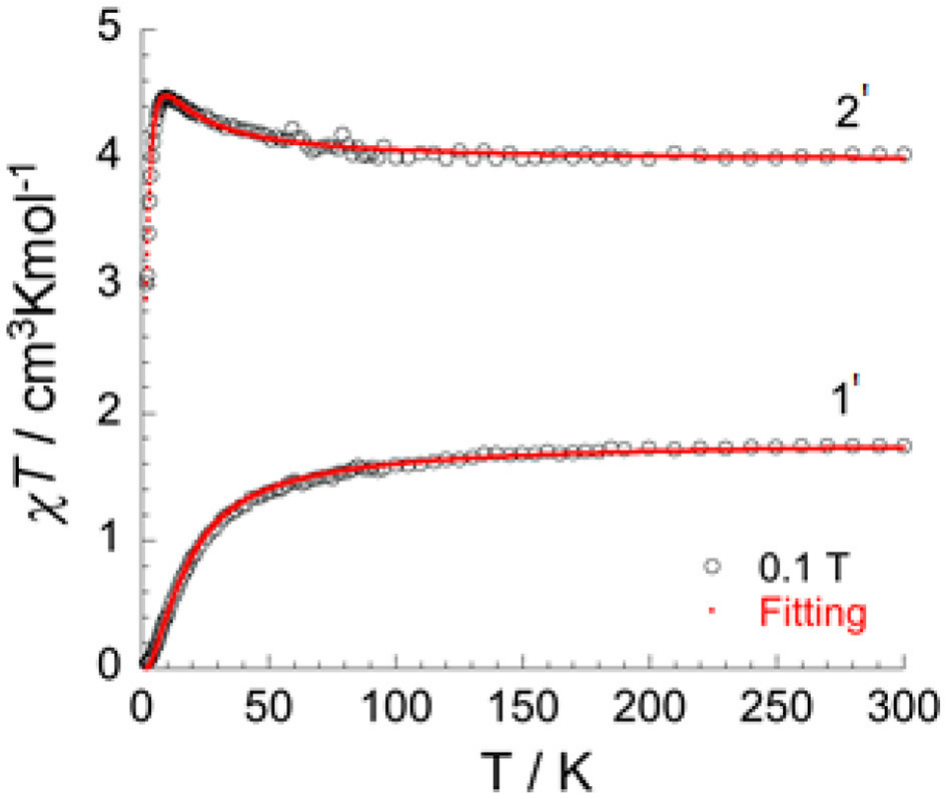
Temperature dependence of χ*T* under 0.1 T applied field for [${\text{V}}_{2}^{\text{III}}$(dpt)_2_Cl_4_]·H_2_O (1') and [${\text{Cr}}_{2}^{\text{III}}$(dpt)_2_Cl_4_]·H_2_O (2'), with the fitted data using Equation (1) (red line).

In order to isolate bulk quantities of desired crystalline products **1** and **2** the reaction mixture was filtered and washed with water. Whilst the single crystal measurements remained unchanged, subsequent microanalysis of the bulk samples shows the presence of water (see experimental). These hydrates of **1** and **2** are referred to as [${\text{V}}_{2}^{\text{III}}$(**dpt**)_2_Cl_4_]·H_2_O (**1'**) and [${\text{Cr}}_{2}^{\text{III}}$(**dpt**)_2_Cl_4_]·H_2_O (**2'**). The χ*T* product vs. temperature under an applied magnetic field of 0.1 T (Figure 7), and the field dependence of magnetization at low temperatures (**Figures S1a–c, S2**), of **1'** and **2'** have been studied.

With the Van Vleck equation (Van Vleck, 1932; Kambe, 1950) an analytical expression of the magnetic susceptibility can be established (Eduok et al., 1983) for the two dimers **1'** and **2'**:

(1) $$\begin{array}{r} \chi_{\text{M}}=\frac{2Ng^{2}\beta^{2}}{kT}\times \frac{e^{2J/kT}+5e^{6J/kT}}{5e^{6J/kT}+3e^{2J/kT}+1}\text{ for }\left(1^{\text{′}}\right) \end{array}$$

(2) $$\begin{array}{r} \chi_{\text{M}}=\frac{2Ng^{2}\beta^{2}}{kT}\times \frac{14e^{12J/kT}+5e^{6J/kT}+e^{2J/kT}}{7e^{12J/kT}+5e^{6J/kT}+3e^{2J/kT}+1}\text{ for }\left(2^{\text{′}}\right) \end{array}$$

*J* represents the exchange interactions in the dimer between the two M^III^ ions in the dinuclear, double triazole bridged, V^III^ dimer **1'** and isostructural Cr^III^ dimer **2'**. With the incorporation of intermolecular interactions (*zJ*) into this equation, the fit leads to the following parameters: *g* = 2.05(0), *J/k*_*B*_ = 1.60(1) K, *zJ/k*_*B*_ = −0.13(1) K for the Cr^III^ dimer **2'** and *J/k*_*B*_ = −7.8(1) K and *g* = 1.89(1) for the V^III^ dimer **1'**.

**Figure 8:**
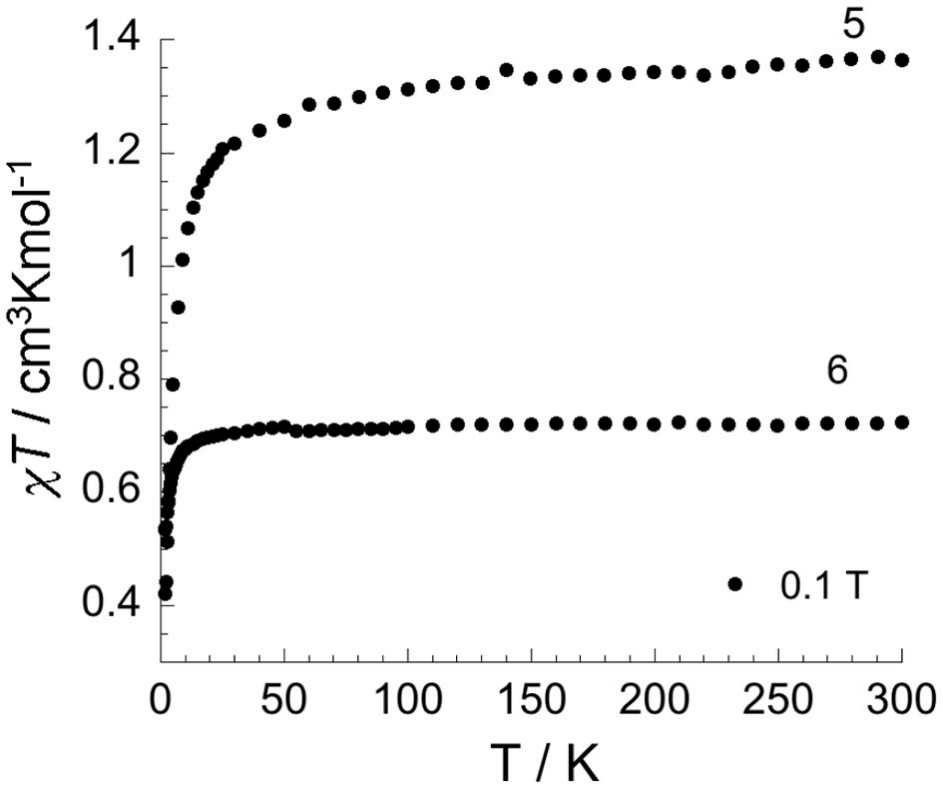
Temperature dependence of χ*T* under 0.1 T applied field for two differently aged samples of [${\text{V}}_{2}^{\text{III}}$(*N*^1^-pydpt)_2_Cl_6_]·2MeCN 4: partially oxidized sample [(V^IV^O)_0.84_(V^III^)_1.16_(*N*^1^-pydpt)_2_Cl_5.16_]·0.84H_2_O·1.16MeCN (5) and fully oxidized sample [(V^IV^O)_2_(*N*^1^-pydpt)_2_Cl_4_] (6) (aged 14 months stored in air) in which complete oxidation to V^IV^V^IV^ has occurred.

The χ*T* product for **1'** is 1.75 cm^3^ K mol^−1^ at room temperature, which is in good agreement for two V^III^ ions (d^2^, *S* = 1, *g*-value = 1.87). On lowering the temperature, the χ*T* product decreases slowly until 70 K and then decreases more rapidly to reach 0.04 cm^3^ K mol^−1^ at 1.8 K (Figure 8). This type of behavior is typical for an antiferromagnetic interaction between the two metal ions with a corresponding total spin ground state of zero (*S*_*T*_ = 0) (Figure S1a). The *M* vs. *H* plot (Figure S1c) confirms the *S*_*T*_ = 0 ground state, with the magnetization showing only a very slow and *quasi*-linear increase with the applied field.

Fitting the experimental χ*T* vs. *T* data for **1'**, above 20 K, to a Curie-Weiss law gives a Curie constant of 2.04 cm^3^ K mol^−1^ and a Weiss constant of −23.4 K (Figure S1b). The negative Weiss constant confirms the antiferromagnetic interaction between the V^III^ ions. Alternatively, the data can be modeled by using the spin Hamiltonian for a dimer with two exchange-coupled V^III^ ions, *H*_*ex*_ = −2*J S*_1_ *S*_2_. The application of the van Vleck equation (Hodgson, 1975; Crawford et al., 1976) makes it possible to determine an analytical expression of the magnetic susceptibility (Equation S1) (Scaringe et al., 1977). The fitting parameters are *g* = 1.89(1) and *J/k*_*B*_ = −7.8(1) K = 5.42 cm^−1^. The g value is in line with what is expected for V^III^ compounds, where the individual g values can vary from 1.83 to 2.03 (Krzystek et al., 2015).

The χ*T* product for **2'** is 4.02 cm^3^ K mol^−1^ at room temperature, which is in line with the expected value for two Cr^III^ ions (d^3^, *S* = 3/2, *g*-value = 2.07). In contrast to the behavior for **1'**, on cooling the χ*T* product for **2'** steadily increases to reach a maximum of 4.48 cm^3^ K mol^−1^ at 10 K, before it drops rapidly to reach a value of 3.01 cm^3^ K mol^−1^ at 1.8 K (Figure 7). This behavior is consistent with ferromagnetic interactions between the two Cr^III^ ions. As for **1'**, the spin Hamiltonian *H*_*ex*_ = −2*J S*_1_ *S*_2_ and Equation (S1) can be applied to fit the data for the two exchange-coupled ions in **2'**, giving *g* = 2.05(0) and *J/k*_*B*_ = +1.60(1) K = +1.11 cm^−1^ (ferromagnetic), as well as weak intermolecular interactions *zJ/k*_*B*_ = −0.13(1) K = −0.09 cm^−1^. The ferromagnetic coupling observed in complex **2'** is quite unusual, as whilst in principle it should be possible to modify the electronic states of dichromium(III) systems such that the nature of the coupling changes from antiferromagnetic to ferromagnetic, very few examples of ferromagnetically coupled dinuclear chromium(III) compounds are known (Døssing, 2014). Following on from their seminal work establishing a linear relationship between the Cu-O-Cu bridging angle and the size of the magnetic coupling for a series of hydroxo-bridged copper(II) complexes (Hodgson, 1975; Crawford et al., 1976), Hodgson and Hatfield reported in 1977 what appears to be the first example of a ferromagnetically coupled chromium(III) dimer, sodium di-μ-hydroxo-*bis*[*bis*(malonato)chromate(III)] pentahydrate (Scaringe et al., 1977). The exchange interaction for this doubly hydroxide bridged compound (*J* = +1.08(2) cm^−1^) is small but consistent with weak ferromagnetic coupling between the Cr^III^ centers. O'Connor et al. reported in 1984 that the dinuclear complex [Cr^III^(L-histidine)(OH)]_2_ shows weak ferromagnetic coupling between the doubly hydroxide bridged Cr^III^ centers (*J*/*k* = +0.15 K = +0.10 cm^−1^) (Eduok et al., 1983). Clearly the provision of double hydroxide bridges can lead to ferromagnetic coupling of the spins in polymetallic Cr^III^ complexes (Morsing et al., 2014) and our present study shows that this can also be facilitated by providing double 1,2,4-triazole bridges in order to provide the required geometry for what seems to be the largest ferromagnetic superexchange interaction, of +1.2 cm^−1^, so far observed in this type of dinuclear Cr(III) system.

The χ_*M*_*T* product vs. temperature for the partially oxidized product of **4**, [(V^IV^O)_0.84_(V^III^)_1.16_(***N***^**1**^-**pydpt**)_2_Cl_5.16_]· 0.84H_2_O·1.16MeCN (**5**) under an applied magnetic field of 0.1 T is shown in Figure 8. The data for **5** are consistent with one of the two V^III^ centers having been oxidized to V^IV^ given that the χ*T* product at room temperature is 1.36 cm^3^Kmol^−1^ which is in line with the theoretical value of 1.375 cm^3^Kmol^−1^ for one V^III^ (d^2^, *S* = 1) and one V^IV^(d^1^, *S* = 1/2). On lowering the temperature, the χ*T* product decreases slowly but steadily until 50 K, at which point it drops rapidly, to 0.42 cm^3^Kmol^−1^ at 1.8 K. Given the large V···V separation this sudden drop is more likely attributable to zero-field-splitting (ZFS) effects than to intramolecular antiferromagnetic interactions. The field-dependence of the magnetization (Figure S3) increases almost linearly up to 7 T, where it reaches 2.04 μ_B_, not showing any sign of saturation. There is no out-of-phase (χ”) signal.

A 14 month old sample of **4** in which full oxidation to V^IV^V^IV^ has occurred, **6**, was also investigated (Figure 8). The χ*T* product at room temperature for **6** is 0.72 cm^3^Kmol^−1^ which is in line with the expected value of 0.75 cm^3^Kmol^−1^ for two oxidized V^IV^ ions. On lowering the temperature, the χ*T* product is almost constant down to 80 K below which it drops rapidly to 0.53 cm^3^Kmol^−1^ at 1.8 K, most likely due to ZFS effects as seen for **5**. The field-dependence of the magnetization at low temperatures (Figure S4a) shows an initial rapid increase for fields up to 3 T, followed by a slightly slower increase up to 7 T, where it reaches 1.78 μ_B._ The non-superposed reduced magnetization curves (Figure S4b) are consistent with the presence of magnetic anisotropy and/or the population of low-lying excited states. As magnetic anisotropy might be present, the temperature dependence of the *ac* susceptibility under zero *dc* field was probed, but as for **5**, no out-of-phase signal was observed for **6**.

As noted above, the magnetic measurements on the two air aged samples of **4** indicate that partial (**5**) or complete (**6**) oxidation of V^III^ to V^IV^ has occurred. This was confirmed for **5** by X-ray crystallography on single crystals after prolonged storage under ambient conditions, which showed that they had converted to [(V^IV^O)_0.84_(V^III^)_1.16_(***N***^**1**^-**pydpt**)_2_Cl_5.16_]· 0.84H_2_O·1.16MeCN **5** (Figure 7, see above), i.e., 42% had oxidised to V^IV^ whilst 58% remained V^III^. In Figure S5 an image of a crystal containing 82% V^III^ with only a 18% oxidation to V^IV^ is shown. In further work it is planned to do a detailed study of the electronic properties, including spectro-electrochemistry as well as femtosecond pump-probe spectroscopy, of (**4**)-(**6**) to determine any valence delocalisation.

No magnetic studies could be performed on compound (**3**) due to lack of material (see experimental).

### Theoretical Study on 1 and 2

Quantum chemical calculations were performed on compounds [${\text{V}}_{2}^{\text{III}}$(**dpt**)_2_Cl_4_] **1** and [${\text{Cr}}_{2}^{\text{III}}$(**dpt**)_2_Cl_4_] **2**. The optimized structures and total energies are given in the Supporting Information (Tables S2 and S3). These complexes are perfect examples of the “two octahedra sharing an edge” situation described in detail in section 9.3 of Kahn's book “Molecular Magnetism” (Kahn, 1993) so the same approach and axis convention was adopted here. Specifically, the geometry optimizations were performed in D_2h_ symmetry, where the C_2_ axes of the dimer are oriented along the coordinate axes (as is standard in that point group), so both metal ions are located on the x-axis, and the Cl^−^-ions in the xz-plane (Figure S6). Hence, in both compounds, the *local* t_2g_-type orbitals are the *local* d_x2−y2_, d_xz_, and d_yz_ orbitals, and they are only partially occupied (electron configurations, d^2^ (S_V_ = 1) and d^3^ (S_Cr_ =3/2), respectively) so are responsible for the magnetic properties.

In the case of **1**, two electrons locally coupled to a triplet state S_V_ = 1 are located at each V ion. The relative energy of the three different possible occupations of pairs of local t_2g_-type orbitals were obtained by calculations on the dimer where the same d-orbitals were occupied at both V-sites (Figure S6). In Table S4 the energies of the three resulting quintet states are presented, and the occupancies of the d-orbitals are given in Table S5. In the ground quintet state, the d_yz_ and d_x2−y2_ orbitals are singly occupied at each V^III^ center [labeled the V(1) state]. In the higher energy quintet states, labeled V(2) and V(3), both metal centers are in an excited state. As a consequence of the relatively weak exchange coupling (see below) between the metal centers, the amount of energy required for a local excitation amounts to half of the energy difference to the ground state so is over 2,000 cm^−1^ (Table S4). Therefore, it can be concluded that these states cannot play any role in terms of understanding the observed magnetic properties.

**Figure 9:**
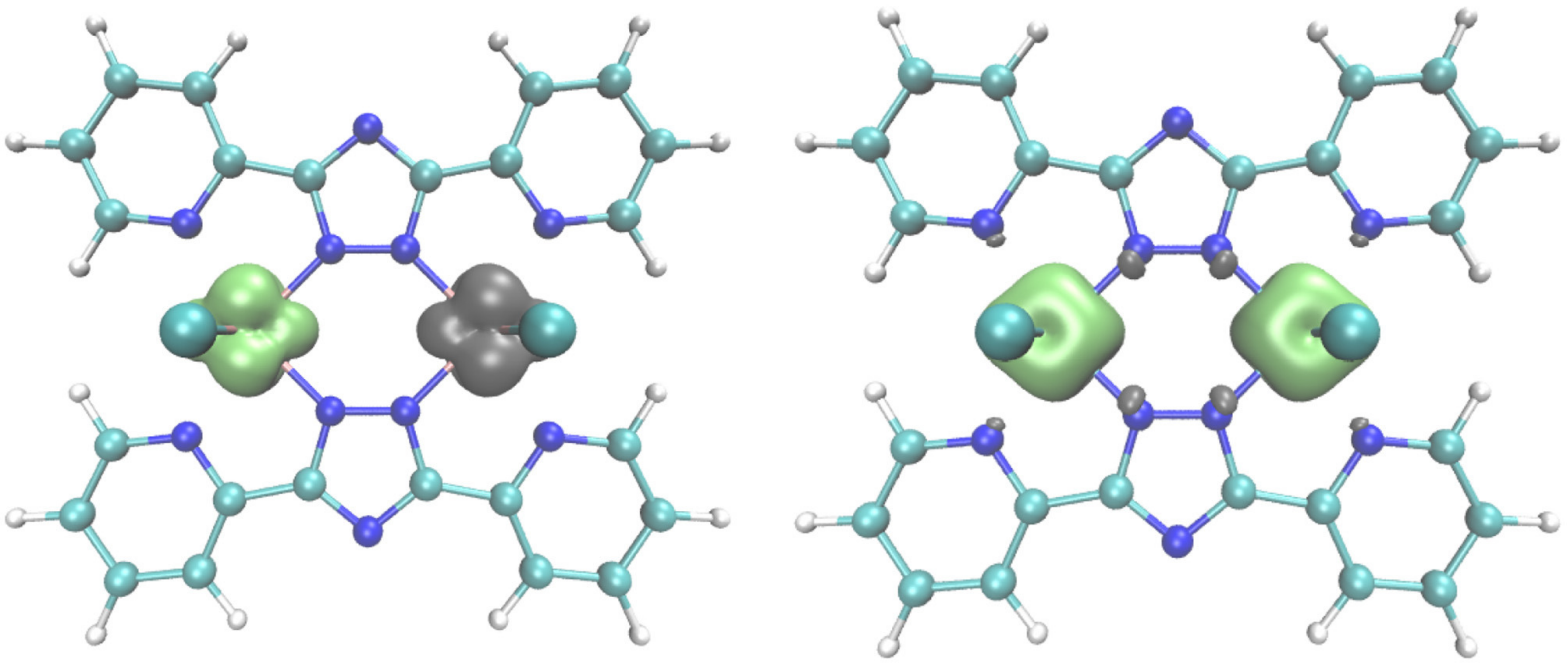
Spin density plots for the the broken symmetry state of the antiferromagnetically coupled vanadium compound (1) (left), and the high spin state (S = 3) of the ferromagnetically coupled chromium compound (2) (right), obtained with the B3-LYP functional and TZVPP basis set. Contour value for the spin density is 0.008.

For **2**, there is only one possible configuration, as all three t_2g_-type orbitals at each of the d^3^ Cr^III^ centers are singly occupied (S_Cr_ = 3/2). In Figure 9 spin densities are shown for the lowest DFT states of (1) and (2). Figure S7 shows the corresponding excited spin states.

In the respective dinuclear complexes, these orbitals were considered in the calculation of the coupling constants. Applying the broken symmetry approach (see experimental section for details), density functional theory (DFT) calculations yielded a weak antiferromagnetic coupling J/k_b_ = −13.0 K for **1** and an even smaller ferromagnetic coupling of J/k_b_ = +1.8 K for **2** (Table S6). Both values are in reasonable agreement with the parameters obtained by fitting to the experimentally observed magnetic data (see above, and SI: J/k_b_ = −7.8 K for **1** and J/k_b_ = +1.6 K for **2**).

For compound **2**, the coupling constant was recalculated by the multi-configuration coupled electron pair approach (MCCEPA) based on a complete active space self-consistent field (CASSCF) wave function (Table S7). Again, a small ferromagnetic coupling, J/k_b_ = +0.7 K, was obtained, consistent with the experimental data.

The change in sign of the magnetic exchange coupling between the ${\text{V}}_{2}^{\text{III}}$ (**1**) and the ${\text{Cr}}_{2}^{\text{III}}$ (**2**) compounds can be rationalized by comparison of the possible ferromagnetic and antiferromagnetic coupling pathways of different orbital pairs. For symmetry reasons an antiferromagnetic coupling by direct or superexchange is only possible between pairs of the same type of orbital, while all pairs of orbitals contribute to the ferromagnetic coupling of the two centers (see Table 9.1 in Kahn's book; Kahn, 1993).

The t_2g_-type orbitals in both complexes look rather similar (Figure S6). Comparing **1** and **2**, the Cr-3d-orbitals should be a bit more compact (lower coupling) because of the higher atomic number of the Cr nucleus. This is partly compensated by the shielding from the additional electron in the d-shell and slightly shorter distances to the coordinating N and the second metal center. Therefore, it can be assumed that the orbital contributions to the coupling constants are identical in both compounds. Using this assumption it was possible to extract the couplings between the pairs of *identical* t_2g_ type d-orbitals, J(d_yz_*d*_yz_), J(d_xz_*d*_xz_),and J(d_x2−y2_*d*_x2−y2_), in each case considering only the sum of the ferromagnetic and antiferromagnetic parts, and then adding an average value J_f⊥_ for the ferromagnetic coupling between the pairs of *different* t_2g_-type orbitals. In Table S6, the results of the broken symmetry calculations corresponding to the three calculated quintet states of **1** as well as for the septet state of **2** are given. Furthermore, the set of linear equations for the determination of the coupling constants involving the various different orbitals and the corresponding values of the constants are given. The J(d_yz_*d*_yz_)/*k*_b_, J(d_xz_*d*_xz_)/*k*_b_ and J(d_x2−y2_*d*_x2−y2_)/*k*_b_ coupling constants (−70.9, −68.6, and −49.4 K) are antiferromagnetic, with J(d_x2−y2_*d*_x2−y2_) slightly smaller than J(d_yz_*d*_yz_) and J(d_xz_*d*_xz_). The ferromagnetic coupling, J_f⊥_/k_b_= +34.2 K, is significantly smaller than all of these. Summing up all contributions to the total values of J (see equation 9.2.3 in Ref. 49b), an antiferromagnetic coupling results in case of **1**, where the total coupling constant is given by J/k_b_=1/4(J(d_yz_*d*_yz_)/*k*_b_ +J(d_x2−y2_*d*_x2−y2_)/*k*_b_ +2J_f⊥_/k_b_) = −13.0 K = −9.0 cm^−1^, whereas for **2** a ferromagnetic coupling dominates, where J/k_b_ =1/9(J(d_yz_*d*_yz_)/*k*_b_+*J*(*d*_xz_*d*_xz_)/*k*_b_ +*J*(*d*_x2−y2_*d*_x2−y2_)/*k*_b_ +6*J*_f⊥_/*k*_b_) = +1.8 K = +1.2 cm^−1^.

## Conclusion

A range of vanadium and chromium complexation reactions using the three ligands 3,5-di-2-pyridyl-4*H*-1,2,4-triazole (H**dpt**), 4-amino-3,5-di(2-pyridyl)-4*H*-1,2,4-triazole (**adpt**) and 2,2'-(4-(pyridin-4-yl)-4,5-dihydro-3*H*-1,2,4-triazole-3,5-diyl)dipyridine (***N***^**4**^-**pydpt**) were performed. As a result of deamination and rearrangements reactions during the solvothermal synthesis only complexes of **dpt**^**−**^ and ***N***^**1**^-**pydpt** were obtained. Clearly these solvothermal conditions facilitate a range of ligand modifications, with deamination of **adpt** to **dpt**^**−**^, and rearrangement of ***N***^**4**^-**pydpt** to ***N***^**1**^-**pydpt**, observed.

The resulting family of complexes, **1**-**5**, represent the first examples of chromium and vanadium complexes of **Rdpt** or **dpt**^**−**^ ligands. They have been structurally and magnetically characterized. The dinuclear vanadium(III) complex (**4**) exhibits a new bridging topology, and the trinuclear chromium complex (**3**) is the first example of a triangular arrangement for an **Rdpt** or **dpt**^**−**^ complex.

To the best of our knowledge, the dinuclear doubly-triazole-bridged chromium(III) complex **2** is the first example of ferromagnetic coupling in a Cr^III^ dimer bridged by any ligand other than hydroxide. Theoretical studies indicate that this is the result of a compensation of a weak antiferromagnetic coupling by super-exchange or direct exchange coupling with the ferromagnetic exchange integrals between the magnetic orbitals of the two centers.

## Experimental Details

### Experimental Methods and Materials

Due to the air-sensitivity of the metal salts to oxidation to higher oxidation states all procedures were carried out under an inert gas atmosphere using a glovebox, glovebag or standard Schlenk technique. The solvents used were dried prior to use. Acetonitrile was pre-dried by distillation from calcium hydride and then by distillation from phosphorous pentoxide before it was stored over molecular sieves (3 Å). Dichloromethane was distilled from phosphorous pentoxide and stored over molecular sieves (4 Å). Alternatively, the solvents used were dried by a Pure Solv MD-6 solvent purification system from Innovative Technology. Chromium metal (99.999%), sodium acetate and vanadium(III) chloride were purchased from Sigma Aldrich, Acros or Fluka and used as received. The ligand 4-amino-3,5-di(2-pyridyl)-4H-1,2,4-triazole (**adpt**) was either purchased from Sigma-Aldrich or synthesized as reported earlier (see also the SI) (White and Brooker, 2013). 4-Pyridyl-3,5-di-2-pyridyl-4*H*-1,2,4-triazole (***N***^4^-**pydpt**) was synthesized as reported earlier (Klingele and Brooker, 2004). Chromium(II) acetate (Herzog et al., 1975a), chromium(II) sulfate (Herzog et al., 1975b) and cobalt pivalate Co_2_(H_2_O)(O_2_CCMe_3_)_4_(HO_2_CCMe_3_)_4_ (Aromí et al., 2003) were synthesized according to the literature methods. For the solvothermal reactions, a 20 mL metal autoclave with a floating Teflon-insert was used, similar to the commercially available acid digestion vessels from Parr Instruments.

Infrared spectra were recorded from KBr-pellets in the range 4,000–400 cm^−1^ with a Perkin-Elmer Spectrum BX FT-IR spectrophotometer (see Supplementary Information, Figure S5). Elemental analyses were either carried out by the Campbell Microanalytical Laboratory at the University of Otago or with a Vario EL spectrometer. X-ray data were collected with a Bruker SMART Apex CCD or Stoe IPDS II diffractometers using graphite-monochromated Mo-Kα radiation (λ = 0.71073 Å) or a Rigaku Oxford Diffraction SuperNova E diffractometer using graphite-monochromated Cu-Kα radiation (λ = 1.54184 Å). Structure solution by direct methods, and full matrix least squares refinement against F^2^ was carried out using SHELXL-2018 (Sheldrick, 2015). Crystals were transferred from the mother liquor into perfluorinated polyether oil to avoid solvent loss before data collection. The magnetic susceptibility measurements were obtained with a Quantum Design SQUID magnetometer MPMS-XL. This magnetometer works between 1.8 and 400 K for *dc* applied fields ranging from −7 to 7 T. Measurements on V_2_(dpt)_2_Cl_4_]·H_2_O (**1'**), ${\text{Cr}}_{2}^{\text{III}}$C_24_H_16_N_10_Cl_4_·H_2_O (**2'**), and partially oxidized [(V^IV^O)_0.84_(V^III^)_1.16_(***N***^**1**^-**pydpt**)_2_Cl_5.16_]·0.84H_2_O·1.16MeCN (**5**) and fully oxidized sample (**6**), were performed on dried polycrystalline samples. The samples used had been stored in air which can result in (a) adsorption of water as indicated by results of elemental analysis (**1'**, **2'**), or (b) in partial or full oxidation (**5**, **6**). *Ac* susceptibility measurements were measured with an oscillating *ac* field of 3 Oe and *ac* frequency at 1,000 Hz, but it is worth noting that no out-of-phase *ac* signal was detected. The magnetic data were corrected for the sample holder and the diamagnetic contribution.

All DFT calculations were performed with the program package TURBOMOLE (Balasubramani et al., 2020). The geometries were obtained under the assumption of D_2h_ symmetry for the high spin states, S = 2 for **1** and S = 3 for **2** using density functional theory (DFT) with the BP86 functional (Becke, 1988; Grummt et al., 2007) and a def2-SVP basis set (Weigend et al., 1998). The exchange coupling constants J were obtained with the B3LYP (Lee et al., 1988) functional and a TZVPP basis (Weigend et al., 1998) set by the broken symmetry approach (Yamaguchi, 1975; Noodleman, 1981; Kizashi et al., 1986; Noodleman and Davidson, 1986) from the energy difference of a high spin calculation E(HS) and a calculation on an electronic state where the unpaired electrons at one metal center are flipped to opposite spin. This state has the energy E(BS) and is called broken symmetry state because it is not an eigenfunction to the S^2^ operator but a linear combination of different spin multiplicities. The exchange coupling constant is then given by J = –(E(HS)-E(BS))/(4S_a_S_b_) where S_a_ and S_b_ are the spins of the different metal centers.

Furthermore, we performed multi-reference calculations with the Bochum program package on compound **2**. Starting from a restricted open shell calculation on the high spin state, a valence configuration interaction calculation was performed with the complete active space self-consistent field (CASSCF) program of Meier and Staemmler (1989) and multi configuration coupled electron pair approach (MCCEPA) calculations (Fink and Staemmler, 1993) were performed. The active space contained the t_2g_-type orbitals of both metal centers. In the correlation treatment the active orbitals and the π-orbitals of the bridge were included. Here, the energies of the septet state, E(7), and the quintet state, E(5), are calculated and J is given by J =-(E(7)–E(5))/6. In the multi-reference calculations, the chromium atoms are equipped with a Wachters basis set (Wachters, 1970) 15s9p5d contracted to 10s6p4d and equipped with an f-function (1.147), the ligands with Huzinaga basis sets. The pyridyl rings are equipped with double zeta basis sets (C and N: 7s5p contracted to 4s2p; H: 3s contracted to 2s), Cl: 11s7p basis contracted to 7s5p, in the triazole ring N: 11s7p contracted to 7s5p +d(1.0) and C: 9s5p contracted to 6s3p+d(0.8).

### Synthesis

#### [${\text{V}}_{2}^{\text{III}}$(dpt)_2_Cl_4_] (1)

Under an inert gas atmosphere, the Teflon-liner of a 20 mL autoclave was loaded with **adpt** (111.4 mg, 0.45 mmol), VCl_3_ (156.8 mg, 0.97 mmol), 6 mL acetonitrile and 5 mL dichloromethane. The solvothermal reaction at 150°C for 45 h followed by slow cooling to room temperature over 6.5 h resulted in orange needle single crystals of [V^III^(**dpt**)Cl_2_]_2_ suitable for X-ray crystallography, and a small amount of an off-white precipitate. Under ambient conditions the crystals were washed with distilled water to remove this precipitate and then air-dried. Yield 31.3 mg, (19.7%). Anal. calc. for [${\text{V}}_{2}^{\text{III}}$(**dpt**)_2_Cl_4_]·H_2_O (**1'**): C_24_H_18_Cl_4_V_2_N_10_O (MW: 706.17): C 40.82, H 2.57, N 19.84; found C 40.66, H 2.50, N 20.06. IR (KBr): $\overset{\sim }{\text{v}}$ = 1653 (vw), 1613 (vs), 1613 (vs), 1501 (w), 1468 (vs), 1437 (w), 1417 (vs), 1401 (sh), 1340 (s), 1287 (w), 1257 (m), 1226 (w), 1204 (s), 1158 (m), 1138 (w), 1147 (sh), 1091 (w), 1068 (w), 1054 (s), 1023 (s), 997 (w), 964 (vw), 897 (vw), 801 (s), 757 (vs), 749 (sh), 723 (vs), 702 (w), 431 (s), 645 (s), 486 (m), 456 (s) cm^−1^. Interestingly, when reacting the deaminated ligand (H**dpt)** with VCl_3_ crystals of **1** were obtained, but in this case they could not be separated from a brown precipitate, so the above route, with *in situ* deamination, is superior.

#### [${\text{Cr}}_{2}^{\text{III}}$(dpt)_2_Cl_4_] (2)

Under an inert gas atmosphere, the Teflon-liner of a 20 mL autoclave was loaded with **adpt** (50 mg, 0.21 mmol), Cr_2_(OAc)_4_(H_2_O)_2_ (50 mg, 0.13 mmol), Co_2_(piv)_4_(Hpiv)_4_ (50 mg, 0.05 mmol), 6 mL acetonitrile and 5 mL dichloromethane. The solvothermal reaction at 150°C for 45 h, followed by followed by slow cooling to room temperature over 6.5 h gave dichroic red-green single crystals of [${\text{Cr}}_{2}^{\text{III}}$(**dpt**)_2_Cl_4_] suitable for X-ray crystallography and a light gray colored precipitate. Under ambient conditions the crystals were washed with water and then air-dried. Yield: 11.6 mg, (15.6%). Anal. calc. for [Cr_2_(**dpt**)_2_Cl_4_]·H_2_O (**2'**): C_24_H_18_Cl_4_Cr_2_N_10_O (MW 708.28): C 40.70, H 2.56, N 19.78; found C 40.68, H 2.70, N 19.50. IR (KBr): $\overset{\sim }{\text{v}}$ = 3411 (vw), 3065 (vw), 2962 (vw), 2922 (vw), 2011 (vw), 1850 (vw), 1655 (w), 1614 (s), 1569 (sh), 1503 (w), 1470 (s), 1418 (vs), 1405 (sh), 1352 (s), 1289 (w), 1265 (sh), 1257 (w), 1235 (w), 1157 (s), 1104 (vw), 1091 (w), 1071 (w), 1023 (s), 998 (w), 964 (vw), 896 (w), 895 (w), 797 (vs), 756 (s), 748 (sh), 721 (vs), 702 (w), 647 (s), 493 (w), 463 (s), 434 (s) cm^−1^. It should be noted that the Cr_2_(OAc)_4_(H_2_O)_2_ complex was produced in house following a literature procedure (Herzog et al., 1975a) involving concentrated HCl, which may provide the source of chloride to this reaction. The magnetically investigated sample came from a cobalt free synthesis which produced a lower yield when substituting Co_2_(piv)_4_(Hpiv)_4_ with pivalic acid. It appears under solvothermal conditions the Co_2_(piv)_4_(Hpiv)_4_ slowly releases the necessary pivalic acid, giving a slower decrease in pH.

#### [${\text{Cr}}_{3}^{\text{III}}$(dpt)_3_Cl_6_]·1¾MeCN·¼DCM (3)

To obtain crystals of trinuclear complex **3** the following synthetic route, starting from **adpt**, is preferred. Under an inert gas atmosphere, the Teflon-liner of a 20 mL autoclave was loaded with **adpt** (50 mg, 0.21 mmol), Cr_2_(OAc)_4_ (50 mg, 0.13 mmol), GdCl_3_ (100 mg, 0.38 mmol), 6 mL acetonitrile and 5 mL dichloromethane, then sealed. The solvothermal reaction at 150°C for 93 h, followed by slow cooling to room temperature over 6.5 h gave dark green single crystals of [${\text{Cr}}_{3}^{\text{III}}$(**dpt**)_3_Cl_6_]·1¾MeCN·¼DCM (**3**), suitable for X-ray crystallography. A few crystals were physically separated as well as possible from a brown gel-like precipitate by washing with water before measuring the IR spectrum. IR (KBr): $\overset{\sim }{\text{v}}$ = 2928 (vw), 2345 (vw), 1615 (vs), 1504 (w), 1471 (s), 1419 (vs), 1384 (w), 1354 (w), 1290 (vw), 1257 (w), 1236 (vw), 1187 (vw), 1158 (w), 1092 (w), 1072 (vw), 1050 (w), 1024 (w), 941 (vw), 926 (vw), 896 (vw), 797 (s), 757 (s), 721 (s), 702 (vw), 647 (w), 492 (vw), 463 (w), 434 (w) cm^−1^.

#### [${\text{V}}_{2}^{\text{III}}$(N^1^-pydpt)_2_Cl_6_]·2MeCN (4) and [(V^IV^O)_0.84_(V^III^)_1.16_(N^1^-pydpt)Cl_5.16_]·0.84H_2_O·1.16MeCN (5)

Under inert gas atmosphere, the Teflon-liner of a 20 mL autoclave was loaded with VCl_3_ (100 mg, 0.64 mmol), ***N***^4^-**pydpt** (151 mg, 0.5 mmol), 6 mL acetonitrile and 5 mL dichloromethane. The solvothermal reaction at 150–100°C for 50 h followed by slow cooling over 5 h gave green single crystals of [V^III^(***N***^**1**^-**pydpt**)Cl_3_]_2_·2MeCN (**4**) suitable for X-ray crystallography, and light gray colored precipitate. Under ambient conditions the crystals were washed repeatedly using small amounts of methanol to remove the precipitate and then air-dried. Yield: 13 mg, (5.5%). Anal. calc. for [${\text{V}}_{2}^{\text{III}}$(***N***^**1**^**-pydpt**)_2_Cl_6_]·2H_2_O (**4'**): C_34_H_24_Cl_6_V_2_N_12_ (MW 951.28): C 42.93, H 2.97, N 17.67, Cl 22.36; found C 42.60, H 2.86, N 17.84, Cl 22.1. IR (KBr): $\overset{\sim }{\text{v}}$ = 434 (m), 454 (m), 539 (m), 617 (s), 633 (vw), 646 (w), 668 (vw), 712 (s), 705 (m), 725 (s), 751 (sh), 759 (s), 800 (s), 807 (sh), 840 (m), 919 (vw), 989 (m), 1013 (s), 1030 (m), 1056 (m), 1098 (sh), 1107 (m), 1153 (w), 1186 (sh), 1194 (w), 1216 (m), 1256 (m), 1290 (m), 1304 (m), 1369 (s), 1423 (vs), 1454 (m), 1473 (s), 1499 (sh), 1507 (vs), 1534 (vw), 1540 (w), 1559 (w), 1570 (vw), 1605 (vs), 1630 (m), 2345 (w), 2370 (w), 2851 (m), 2925 (m), 3070 (s), 3103 (sh) cm^−1^. Green-brown single crystals of **5** were obtained from storing crystals of **4** in air under ambient conditions. There were not enough crystals to measure CHN and IR, therefore only IR was measured. IR (KBr): $\overset{\sim }{\text{v}}$ = 434 (w), 454 (w), 539 (vw), 617 (m), 633 (w), 646 (w), 705 (m), 725 (s), 759 (s), 800 (m), 807 (sh), 840 (m), 919 (vw), 989 (m), 1013 (s), 1030 (m), 1056 (m), 1107 (m), 1153 (w), 1194 (m), 1216 (m), 1256 (m), 1290 (m), 1304 (w), 1369 (s), 1423 (vs), 1454 (m), 1473 (s), 1499 (sh), 1507 (vs), 1534 (vw), 1540 (w), 1559 (w), 1570 (w), 1605 (vs), 1630 (s), 2345 (vw), 2370 (vw), 2925 (vw), 3070 (w), 3103 (w) cm^−1^. Full oxidation from **4** to [(V^IV^O)_2_(***N***^**1**^-**pydpt**)_2_Cl_4_] (**6)** takes several months under ambient conditions, and results in a loss of crystal quality so the structure could not be determined. In an attempt to isolate a bulk sample of the V^III^-V^III^ system, multiple approaches were taken to exclude oxygen at all stages of the reaction. This included using VCl_3_ from both Sigma Aldrich and Alfa Aesar, using two different glove boxes at Karlsruhe Institute of Technology, using freshly dried dichloromethane and acetonitrile, using several different autoclaves, as well as two batches of the triazole ligand. In all cases we observed partial oxidation to V^IV^, with the minimum conversion being 10%. The crystal structure which we obtained of the V^III^-V^III^ compound appears to be serendipitous as bulk sample clearly contains V^IV^ as evidenced by the DC magnetic susceptibility and the vanadyl stretch at 989 cm^−1^ in the IR (Hamilton, 1991).

## Data Availability Statement

The datasets generated for this study can be found in the Crystallographic data available from the CCDC via https://summary.ccdc.cam.ac.uk/structure-summary-form or e-mail: data_request@ccdc.cam.ac.uk as 1853503, 1853504, 1853505, 1853506, 1853507, and 1984490.

## Author Contributions

JR, JK, and AC performed synthesis and standard characterization. YL performed the magnetic measurements. AC and CA performed the crystal structure analyses. KF performed the quantum chemical calculations. AP and SB conceived and supervised the work. All authors contributed to the article and approved the submitted version.

## Conflict of Interest

The authors declare that the research was conducted in the absence of any commercial or financial relationships that could be construed as a potential conflict of interest.
