# Supplementary material for: Di- and Tri-nuclear VIII and CrIII Complexes of Dipyridyltriazoles: Ligand Rearrangements, Mixed Valency and Ferromagnetic Coupling
Source: Front Chem. 2020 Jul 9;8:540. doi: 10.3389/fchem.2020.00540 (PMC7363982; doi:10.3389/fchem.2020.00540)
Supplement: Supplementary file 1 [file Table_1.docx]

Supplementary Material for

**Di- and Tri-nuclear V^III^ and Cr^III^ Complexes of Dipyridyltriazoles: Ligand Rearrangements, Mixed Valency and Ferromagnetic Coupling**

Julia Rinck^1^, Anthony B. Carter^1^, Jonathan A. Kitchen^2,3^, Yanhua Lan^1^, Christopher E. Anson^1^, Karin Fink^4^*, Sally Brooker^2^* and Annie K. Powell^1,4^*

^1^Institute of Inorganic Chemistry, Karlsruhe Institute of Technology, Engesserstrasse 15, 76131 Karlsruhe, Germany.

^2^Department of Chemistry and MacDiarmid Institute for Advanced Materials and Nanotechnology, University of Otago, PO Box 56, Dunedin 9054, New Zealand.

^3^Chemistry, School of Natural and Computational Sciences, Massey University, Auckland 0632, New Zealand

^4^Institute of Nanotechnology, Karlsruhe Institute of Technology, Hermann-von-Helmholtz-Platz 1, 76344 Eggenstein-Leopoldshafen, Germany.

*** Correspondence:**Corresponding Authors
sbrooker@chemistry.otago.ac.nz
karin.fink@kit.edu
annie.powell@kit.edu

1. **Magnetic data**

**Figure S1a:** Dinuclear vanadium(III) complex **1’**. Extrapolation of *χT* at low temperature, indicating a total spin groung state of zero (S_T_ = 0).

**Figure S1b:** Dinuclear vanadium(III) complex **1’**. Fitting of the experimental data of the temperature dependence of *χT* to a Curie-Weiss law above 20 K.

**Figure S1c:** Dinuclear vanadium(III) complex **1’**. Field dependence of the magnetisation at different temperatures.


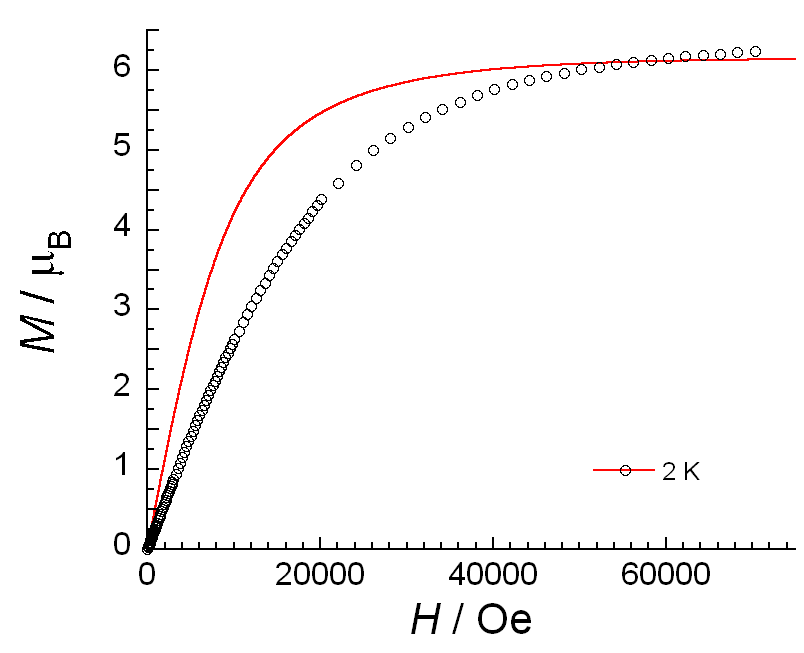


**Figure S2:** Dinuclear chromium(III) complex **2’**. Field-dependence of magnetisation at 2 K, experimental (black dots) and calculated data (red solid line). The theoretical data was obtained using a Brillouin function with S = 3 and g = 2.05.


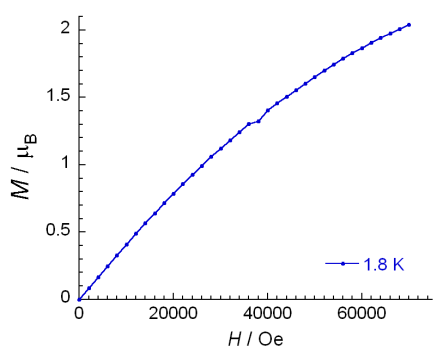


**Figure S3:** Partially oxidized dinuclear vanadium(III/IV) complex **5**. Field-dependence of the magnetization at 1.8 K.


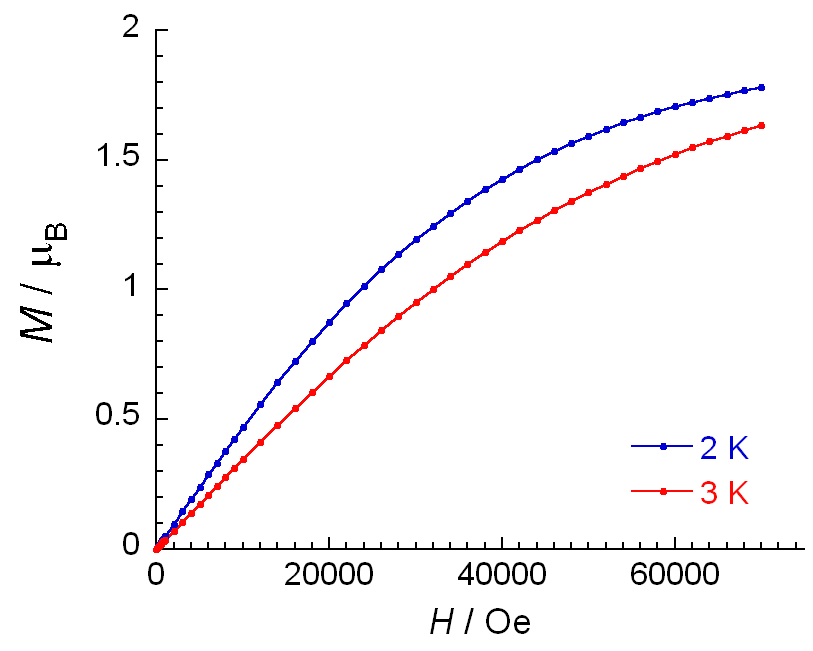


**Figure S4a:** Fully oxidized dinuclear vanadium(IV/IV) complex **6**. Field-dependence of the magnetization at the indicated temperatures.


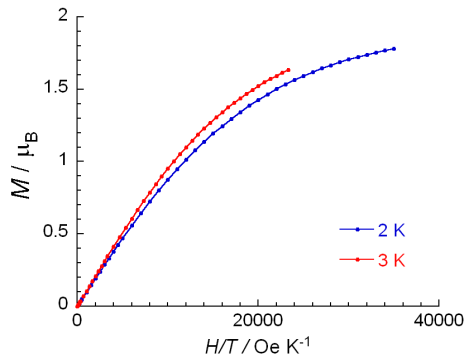


**Figure S4b:** Fully oxidized dinuclear vanadium(IV/IV) complex **6**. Field-dependence of the reduced magnetization, *M* vs *H*/*T*, at the indicated temperatures.

With the Van Vleck equation an analytical expression of the magnetic susceptibility can be established for the two dimers **1’** and **2’**:

$\chi_{\text{M}}=\frac{2Ng^{2}\beta^{2}}{kT}\times\frac{e^{2J/kT}+5e^{6J/kT}}{5e^{6J/kT}+3e^{2J/kT}+1}$ for (**1’**) (equation S1)

$\chi_{\text{M}}=\frac{2Ng^{2}\beta^{2}}{kT}\times\frac{14e^{12J/kT}+5e^{6J/kT}+e^{2J/kT}}{7e^{12J/kT}+5e^{6J/kT}+3e^{2J/kT}+1}$ for (**2’**) (equation S2)

*J* represents the exchange interactions in the dimer between the two M^III^ ions in the dinuclear, double triazole bridged, vanadium(III) dimer **1’** and isostructural chromium(III) dimer **2’**. With the incorporation of intermolecular interactions (*zJ*) into this equation, the fit leads to the following parameters: *g* = 2.05(0), *J/k_B_* = 1.60(1) K, *zJ/k_B_* = -0.13(1) K for the chromium(III) dimer **2’** and *J/k_B_* = -7.8(1) K and *g* = 1.89(1) for the vanadium(III) dimer **1’**.

**Magnetism References**

[S1] J. H. van Vleck, *The Theory of Electric and Magnetic Susceptibility*, *Oxford University Press*, (**1932**); K. Kambe, J. Phys. Soc. Jpn.5 (**1950**) 48.

[S2] C. J. O’Connor, *Progress in Inorganic Chemistry*, **1982**, 29, 203-283.

1. **Crystallographic data**


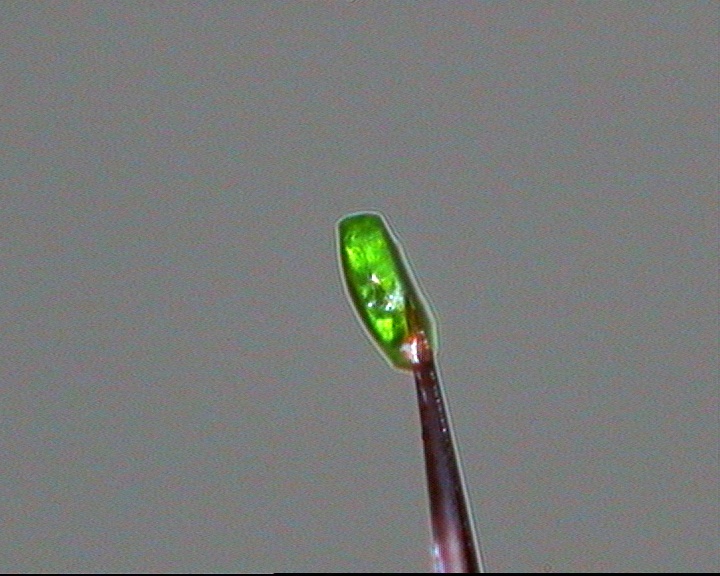


**Figure S5:** Image of a sample of the partially oxidized compound 5 in this crystal the oxidized formular is [(V^IV^O)_0.2_(V^III^)_1.8_(*N*^1^-pydpt)_2_Cl_5.8_].

Table S1: Crystallographic data:

| Compound | [Cr(**dpt**)Cl_2_]_2_  (**1**) | [V(**dpt**)Cl_2_]_2_  (**2**) | [Cr(**dpt**)Cl_2_]_3_·1.75MeCN·0.25CH_2_Cl_2_  (**3**) |  |
| --- | --- | --- | --- | --- |
| Formula | C_24_H_16_Cl_4_Cr_2_N_10_ | C_24_H_16_Cl_4_N_10_V_2_ | C_39.75_H_29.75_Cl_6.50_Cr_3_N_16.75_ |  |
| Formula weight [g mol^-1^] | 690.27 | 688.15 | 1128.48 |  |
| Crystal System | orthorhombic | orthorhombic | monoclinic |  |
| Space Group | P*nnm* | P*nnm* | *P*2_1_/*c* |  |
| Cell constants *a* [Å] | 9.8896(14) | 9.8288(13) | 11.3743(5) |  |
| *b* [Å] | 10.4210(9) | 10.5383(12) | 38.9078(17) |  |
| *c* [Å] | 13.397(2) | 13.259(2) | 11.0105(5) |  |
| α [°] | 90 | 90 | 90 |  |
| β [°] | 90 | 90 | 102.326(1) |  |
| γ [°] | 90 | 90 | 90 |  |
| Volume [Å^3^] | 1380.7(3) | 1373.4(3) | 4760.4(4) |  |
| Z | 2 | 2 | 4 |  |
| T [K] | 150(2) | 150(2) | 100(2) |  |
| *F*(000) | 692 | 688 | 2272 |  |
| *D*_calc_ [g cm^-3^] | 1.66 | 1.664 | 1.575 |  |
| *μ* (Mo-*K*_α_) [mm^-1^] | 1.21 | 1.106 | 1.088 |  |
| Diffractometer | Stoe IPDS II | Stoe IPDS II | Bruker Smart Apex |  |
| Data Measured | 9660 | 8540 | 30217 |  |
| Unique Data | 1724 | 1530 | 9647 |  |
| Unique reflections *R*_int_ | 0.0405 | 0.0401 | 0.0303 |  |
| Reflections with *I* ≥ 2σ(*I*) | 1408 | 1256 | 9052 |  |
| Parameters | 96 | 96 | 624 |  |
| Restraints | 0 | 0 | 10 |  |
| *wR*_2_ (all data) | 0.1414 | 0.149 | 0.1158 |  |
| *R*_1_ [*I* ≥ 2σ(*I*)] | 0.0556 | 0.0573 | 0.0542 |  |
| *S* (all data) | 0.993 | 1.044 | 1.035 |  |
| Biggest diff. peak/hole [Å^3^] | 0.369/-0.996 | 0.745/-0.821 | 0.622/-0.411 |  |

| Compound | [V(pydpt)Cl_3_]_2_·2MeCN  (**4**) | [(V^IV^O)_0.84_(V^III^)_1.16_(pydpt)_2_Cl_5.16_] ·0.84H_2_O∙1.16MeCN (**5**) |
| --- | --- | --- |
| Formula | C_38_H_30_Cl_6_N_14_V_2_ | C_36.32_H_29.16_Cl_5.16_N_13.16_O_1.68_V_2_ |
| Formula weight [g mol^-1^] | 997.34 | 961.65 |
| Crystal System | triclinic | triclinic |
| Space Group | *P*-1 | *P*-1 |
| Cell constants *a* [Å] | 8.853(2) | 8.8302(9) |
| *b* [Å] | 11.014(3) | 11.0282(11) |
| *c* [Å] | 12.255(3) | 12.2533(12) |
| α [°] | 69.267(18) | 67.901(2) |
| β [°] | 70.330(19) | 69.489(2) |
| γ [°] | 73.229(19) | 73.071(2) |
| Volume [Å^3^] | 1032.7(4) | 1017.88(18) |
| Z | 1 | 1 |
| T [K] | 150(2) | 100(2) |
| *F*(000) | 504 | 486 |
| *D*_calc_ [g cm^-3^] | 1.604 | 1.569 |
| *μ* (Mo-*K*_α_) [mm^-1^] | 0.891 | 0.849 |
| Diffractometer | Stoe IPDS II | Bruker SMART Apex |
| Data Measured | 7825 | 7194 |
| Unique Data | 4362 | 4349 |
| Unique reflections *R*_int_ | 0.0635 | 0.0155 |
| Reflections with *I* ≥ 2σ(*I*) | 3402 | 4032 |
| Parameters | 272 | 270 |
| Restraints | 2 | 5 |
| *wR*_2_ (all data) | 0.1896 | 0.1281 |
| *R*_1_ [*I* ≥ 2σ(*I*)] | 0.0675 | 0.0521 |
| *S* (all data) | 1.027 | 1.133 |
| Biggest diff. peak/hole [Å^3^] | 1.807/-1.015 | 0.666/-0.525 |

1. **Data from the quantum chemical calculations**


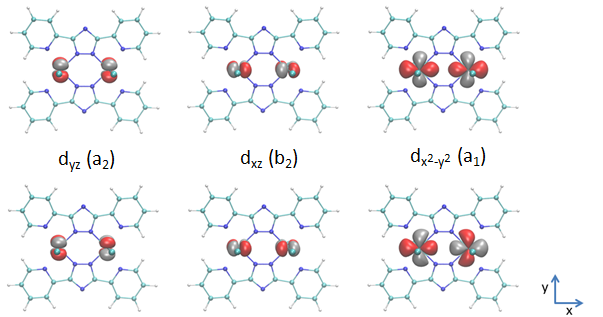


Figure S6: Sketches of the six ±-linear combinations of the magnetic orbitals, the three singly occupied *local* t_2g_ -type orbitals at each d^3^ metal center, in the dinuclear Cr^III^ complex (2), using the literature axis convention (shown).^49b^ The form of the t_2g_-type orbitals is identical in the case of the dinuclear V^III^ complex (1), but only two of the orbitals are singly occupied at each d^2^ metal center in that case.

**
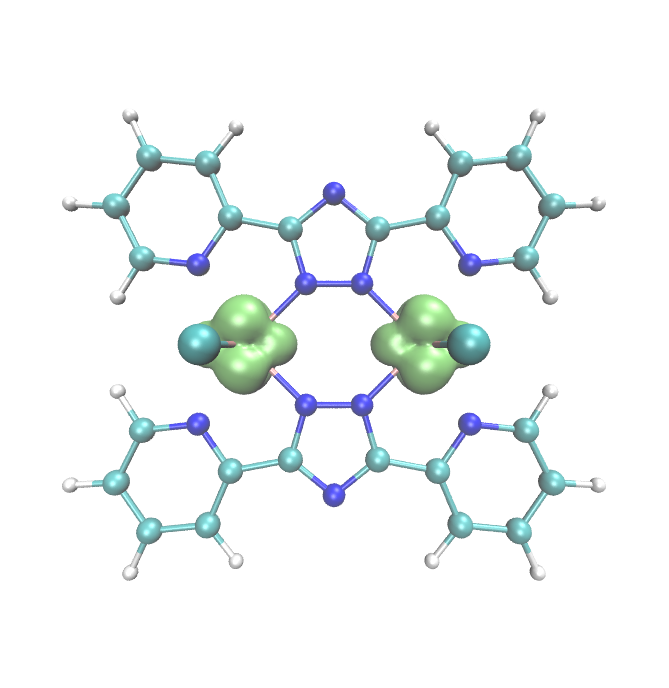

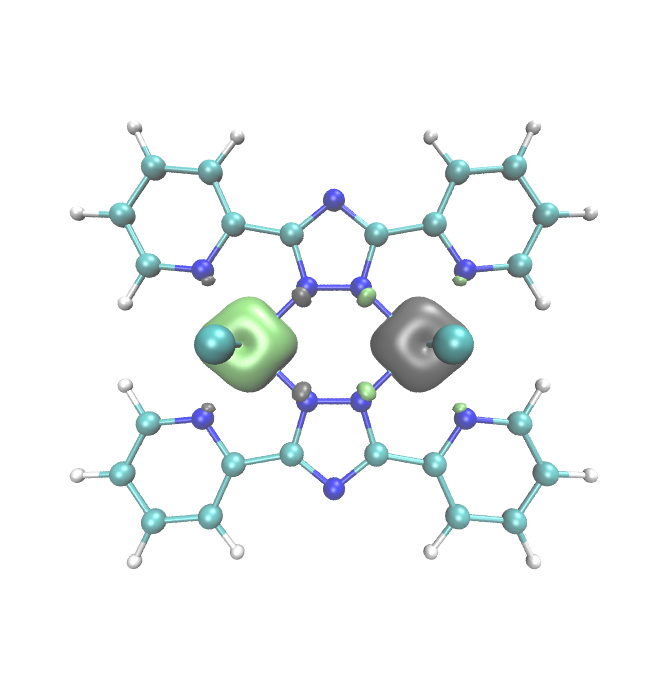
**

Figure S7: Spin density plots for the ferromagnetically coupled vanadium compound (1) (left), and the antiferromagnetically coupled chromium compound (2), obtained with the B3-LYP functional and TZVPP basis set. Contour value for the spin density is 0.008.

**Table S2:** Optimized structure of the dinuclear vanadium(III) complex [V^III^_2_(**dpt**^-^)_2_Cl_4_] (**1**) in xyz format. All coordinates are given in Ångström, the geometry optimizations were performed in the point group D_2h_ with the BP86 functional, a def2-SVP basis set for the high spin state with a total of 4 unpaired electrons across the pair of d^2^ vanadium(III) centres.

56

Energy = -5199.739074100H

V 2.1199836 0.0000000 0.0000000

V -2.1199836 0.0000000 0.0000000

Cl 2.5654522 0.0000000 2.2392581

Cl 2.5654522 0.0000000 -2.2392581

Cl -2.5654522 0.0000000 2.2392581

Cl -2.5654522 0.0000000 -2.2392581

N 0.6702259 1.4520849 0.0000000

N 0.6702259 -1.4520849 0.0000000

N -0.6702259 1.4520849 0.0000000

N -0.6702259 -1.4520849 0.0000000

N 0.0000000 3.6115318 0.0000000

N 0.0000000 -3.6115318 0.0000000

N 3.2536818 1.9086631 0.0000000

N 3.2536818 -1.9086631 0.0000000

N -3.2536818 1.9086631 0.0000000

N -3.2536818 -1.9086631 0.0000000

C 1.0462263 2.7600795 0.0000000

C 1.0462263 -2.7600795 0.0000000

C -1.0462263 2.7600795 0.0000000

C -1.0462263 -2.7600795 0.0000000

C 2.4775956 3.0314364 0.0000000

C 2.4775956 -3.0314364 0.0000000

C -2.4775956 3.0314364 0.0000000

C -2.4775956 -3.0314364 0.0000000

C 3.0264783 4.3265705 0.0000000

C 3.0264783 -4.3265705 0.0000000

C -3.0264783 4.3265705 0.0000000

C -3.0264783 -4.3265705 0.0000000

C 4.4185446 4.4656472 0.0000000

C 4.4185446 -4.4656472 0.0000000

C -4.4185446 4.4656472 0.0000000

C -4.4185446 -4.4656472 0.0000000

C 5.2170956 3.3082016 0.0000000

C 5.2170956 -3.3082016 0.0000000

C -5.2170956 3.3082016 0.0000000

C -5.2170956 -3.3082016 0.0000000

C 4.5899074 2.0551362 0.0000000

C 4.5899074 -2.0551362 0.0000000

C -4.5899074 2.0551362 0.0000000

C -4.5899074 -2.0551362 0.0000000

H 5.1828904 1.1282660 0.0000000

H 5.1828904 -1.1282660 0.0000000

H -5.1828904 1.1282660 0.0000000

H -5.1828904 -1.1282660 0.0000000

H 2.3461390 5.1894331 0.0000000

H 2.3461390 -5.1894331 0.0000000

H -2.3461390 5.1894331 0.0000000

H -2.3461390 -5.1894331 0.0000000

H 4.8797933 5.4646881 0.0000000

H 4.8797933 -5.4646881 0.0000000

H -4.8797933 5.4646881 0.0000000

H -4.8797933 -5.4646881 0.0000000

H 6.3146285 3.3697170 0.0000000

H 6.3146285 -3.3697170 0.0000000

H -6.3146285 3.3697170 0.0000000

H -6.3146285 -3.3697170 0.0000000

**Table S3:** Optimized structure of dinuclear chromium(III) complex [Cr^III^_2_(**dpt^-^**)_2_Cl_4_] (**2**) in xyz format. All coordinates are given in Ångström, the geometry optimizations were performed in the point group D_2h_ with the BP86 functional, a def2-SVP basis set for the high spin state with a total of 6 unpaired electrons across the pair of d^3^ chromium(III) centres.

56

Energy = -5400.649109656 H

Cr 2.0883462 0.0000000 0.0000000

Cr -2.0883462 0.0000000 0.0000000

Cl 2.3492605 0.0000000 2.2652125

Cl 2.3492605 0.0000000 -2.2652125

Cl -2.3492605 0.0000000 2.2652125

Cl -2.3492605 0.0000000 -2.2652125

N 0.6644563 -1.4042154 0.0000000

N 0.6644563 1.4042154 0.0000000

N -0.6644563 -1.4042154 0.0000000

N -0.6644563 1.4042154 0.0000000

N 0.0000000 3.5610377 0.0000000

N 0.0000000 -3.5610377 0.0000000

N 3.2402896 -1.8299366 0.0000000

N 3.2402896 1.8299366 0.0000000

N -3.2402896 -1.8299366 0.0000000

N -3.2402896 1.8299366 0.0000000

C 1.0460088 -2.7102734 0.0000000

C 1.0460088 2.7102734 0.0000000

C -1.0460088 -2.7102734 0.0000000

C -1.0460088 2.7102734 0.0000000

C 2.4790483 -2.9638037 0.0000000

C 2.4790483 2.9638037 0.0000000

C -2.4790483 -2.9638037 0.0000000

C -2.4790483 2.9638037 0.0000000

C 3.0520804 -4.2483404 0.0000000

C 3.0520804 4.2483404 0.0000000

C -3.0520804 -4.2483404 0.0000000

C -3.0520804 4.2483404 0.0000000

C 4.4462710 -4.3627400 0.0000000

C 4.4462710 4.3627400 0.0000000

C -4.4462710 -4.3627400 0.0000000

C -4.4462710 4.3627400 0.0000000

C 5.2260679 -3.1931783 0.0000000

C 5.2260679 3.1931783 0.0000000

C -5.2260679 -3.1931783 0.0000000

C -5.2260679 3.1931783 0.0000000

C 4.5777419 -1.9510852 0.0000000

C 4.5777419 1.9510852 0.0000000

C -4.5777419 -1.9510852 0.0000000

C -4.5777419 1.9510852 0.0000000

H 2.3877321 -5.1234409 0.0000000

H 2.3877321 5.1234409 0.0000000

H -2.3877321 -5.1234409 0.0000000

H -2.3877321 5.1234409 0.0000000

H 4.9243935 -5.3537595 0.0000000

H 4.9243935 5.3537595 0.0000000

H -4.9243935 -5.3537595 0.0000000

H -4.9243935 5.3537595 0.0000000

H 6.3242958 -3.2355356 0.0000000

H 6.3242958 3.2355356 0.0000000

H -6.3242958 -3.2355356 0.0000000

H -6.3242958 3.2355356 0.0000000

H 5.1549184 -1.0152080 0.0000000

H 5.1549184 1.0152080 0.0000000

H -5.1549184 -1.0152080 0.0000000

H -5.1549184 1.0152080 0.0000000

**Table S4:** Energies of the quintet states (S=5) in [V^III^_2_(**dpt**^-^)_2_Cl_4_] (**1**), obtained for single occupancy of the three different pairings of possible *local* t_2g_-type orbitals at each V^III^ centre, which in D_2h_ symmetry belong to different irreducible representations, with all 4 of these d electrons having parallel spins (hence S=5 overall). The occupation numbers are given in Table S5. The results are obtained from DFT calculations with the B3LYP functional and the TZVPP basis set (for more details see the manuscript). The energy differences **ΔE** include local excitations at both centers. The t_2g_ orbitals are shown in Figure 10 and look identical in **1** and **2**. Note that for the d^3^ chromium(III) analogue, dimer **2**, in the septet state (S=7; all 6 of these d electrons having parallel spins) there is only one possible occupation of the t_2g_-type d-orbitals – i.e. with all three of them singly filled at both centers.

**Occupied orbitals at each d^2^ V^III^ Label Energy [H] ΔE/k_b_[K] ΔE/k_b_[cm^-1^]**

**center 1 center2**

d_yz_^1^ d_x2-y2_^1^ d_yz_^1^ d_x2-y2_^1^ V(1) -5200.360949327 0 0

d_xz_^1^ d_x2-y2_^1^  d_xz_^1^ d_x2-y2_^1^  V(2) -5200.339609316 6739 4684

d_xz_^1^  d_yz_^1^  d_xz_^1^  d_yz_^1^  V(3) -5200.312585160 15272 10615

**Table S5:** Natural population analyses (broken symmetry states for complex **1**, septet state for complex **2**). The differences in the occupation numbers between alpha spin and beta spin obtained by a natural population analysis (A.E. Reed, R.B. Weinstock and F. Weinhold, Natural population analysis, *J. Chem. Phys.* 83, 735, **1985**) are given. These clearly confirm that the magnetic orbitals are the t2g-type orbitals (dxz, dyz and dz2-y2 using the axis convention shown in Figure 10), in bold type in the table, in both complexes.

| **center** | **d-orbital** | **V(1)** | **V(2)** | **V(3)** | **Cr** |
| --- | --- | --- | --- | --- | --- |
| **1** | dz2 | -0.13 | -0.09 | -0.06 | 0.06 |
|  | **dxz** | -0.07 | **-0.85** | **-0.84** | **0.86** |
|  | **dyz** | **-0.85** | -0.06 | **-0.84** | **0.85** |
|  | dxy | -0.09 | -0.09 | -0.10 | 0.03 |
|  | **dx2-y2** | **-0.90** | **-0.94** | -0.04 | **0.94** |
| **2** | dz2 | 0.10 | 0.06 | 0.04 | 0.06 |
|  | **dxz** | 0.05 | **0.87** | **0.86** | **0.86** |
|  | **dyz** | **0.87** | 0.05 | **0.86** | **0.85** |
|  | dxy | 0.05 | 0.05 | 0.05 | 0.03 |
|  | **dx2-y2** | **0.91** | **0.95** | 0.03 | **0.94** |

**Table S6:** Exchange coupling constants for [V^III^_2_(**dpt**^-^)_2_Cl_4_] (**1**) and [Cr^III^_2_(**dpt**^-^)_2_Cl_4_] (**2**) obtained from DFT calculations with the B3LYP functional and the TZVPP basis set on the high spin (HS) and broken symmetry (BS) states (for more details see the manuscript). Extrapolation of the different contributions to the total coupling constants:

J(d_yz_d_yz_) = J_f_(d_yz_d_yz_) + J_af_(d_yz_d_yz_)

J(d_xz_d_xz_) = J_f_(d_xz_d_xz_) + J_af_(d_xz_d_xz_)

J(d_x2-y2_d_x2-y2_) = J_f_(d_x2-y2_d_x2-y2_) + J_af_(d_x2-y2_d_x2-y2_)

J_f⊥_ = (d_1i_d_2j_|d_1i_d_2j_) (two electron integral in Mulliken notation) where 1, 2 are the different metal centers and i, j are different local t_2g_ orbitals.

Metal E(HS)[H] E(BS)[H] J/k_b_[K] J/k_b_[cm^-1^]

V (1) -5200.360949302 -5200.361113896 -13.0 -9.0

V (2) -5200.339609316 -5200.339766647 -12.4 -8.6

V (3) -5200.312585160 -5200.312810767 -17.8 -12.4

Cr -5401.277192904 -5401.277142177 +1.8 +1.2

V1: 4*J(V1)/k_b_ **=** J(d_yz_d_yz_)/k_b_ + J(d_x2-y2_d_x2-y2_)/k_b_ + 2J_f⊥_/k_b_ = -52.0 K

V2: 4*J(V2)/k_b_ **=** J(d_xz_d_xz_)/k_b_+ J(d_x2-y2_d_x2-y2_)/k_b_ + 2J_f⊥_/k_b_ = -49.7 K

V3: 4*J(V3)/k_b_ **=** J(d_yz_d_yz_)/k_b_+ J(d_xz_d_xz_)/k_b_ + 2J_f⊥_/k_b_ = -71.2 K

Cr : 9*J(Cr)/k_b_ = J(d_yz_d_yz_)/k_b_+ J(d_xz_d_xz_) /k_b_+ J(d_x2-y2_d_x2-y2_)/k_b_ + 6J_f⊥_/k_b_ = +16.0 K

Where for both complexes K can be converted to cm^-1^ as follows (noting that k_b_=1.38 x 10^-23^ JK^-1^, and that 1 J = 5.04 x 10^22^ cm^-1^):

J(d_yz_d_yz_)/k_b_ = -70.9 K = -49.3 cm^-1^

J(d_xz_d_xz_) /k_b_ = -68.6 K = -47.7 cm^-1^

J(d_x2-y2_d_x2-y2_) /k_b_ = -49.4 K = -34.3 cm^-1^

J_f⊥_/k_b_ = +34.2 K = +23.7 cm^-1^

Note that the Kahn equations (O. Kahn, *Molecular Magnetism*, VCH Publishers Inc., New York, **1993**, section 9.3) are used above, so the orbital contributions are taken with S_1_=S_2_=1/2, i.e. E(HS)-E(BS) is equal to the sum of the couplings of the orbitals, while the total J is determined with S_1_=S_2_=1 for vanadium(III) and S_1_=S_2_=3/2 for chromium(III).

**Table S7:** Exchange coupling constant for [Cr^III^_2_(**dpt**^-^)_2_Cl_4_] (**2**) obtained with the MCCEPA method (for more details see the manuscript).

E(7)[H] E(5)[H] J/k_b_[K] J/k_b_[cm^-1^]

Cr -5386.29000010 -5386.28998668 0.7 0.5

1. **Infrared Spectra**

**Figure S5:** Infrared spectra of [V^III^(**dpt**)Cl_2_]_2_ H_2_O (**1’**) (green), [Cr^III^(**dpt**)Cl_2_]_2_ H_2_O (**2’**) (red), dried crystals of [Cr^III^_3_(**dpt**)_3_Cl_6_]·1¾MeCN·¼DCM (**3**) (blue), [V^III^_2_(***N*^1^**-**pydpt**)_2_Cl_6_]·2MeCN (**4**) (black).

1. **Additional Synthetic Details**

**Hdpt**

Obtained by slight modifications of the literature method**^S3^** for the deamination of 4-amino-3,5-di-2pyridyl-4H-1,2,4-triazole (**adpt**) - but it should be noted that this H**dpt** ligand can also be purchased. In a 50 mL conical flask in a fumehood on boost, 4-amino-3,5-di-2-pyridyl-4H-1,2,4-triazole (**adpt**; 1 g, 4.2 mmol) was dissolved in 10 mL of 5 M nitric acid by stirring at room temperature. An aqueous solution (4 mL) of sodium nitrite (2 g, 28.99 mmol) was cautiously added dropwise. The mixture was stirred for 20 min at room temperature before it was boiled until no more brown gas evolved (caution: fumehood on boost). After cooling down to room temperature, concentrated aqueous ammonia (2.5 mL of 33 %) was added cautiously dropwise to reach a pH of 9. The resulting white solid was filtered and dried in vacuo. The desired product was extracted over 24 h, from the crude solid, using a Soxhlet extractor and toluene (300 mL). The resulting toluene extract was allowed to cool slowly to room temperature, resulting in white needles of H**dpt** which were filtered off and dried in vacuo. Yield: 752.2 mg (80.2 %). Anal. calc. for C_12_H_9_N_5_ (MW 223.24): C 64.56, H 4.06, N 31.37; found C 64.82, H 4.29, N 31.12.

**Reference for deamination**

[S3] Geldard J. F.; Lions F. *J. Org. Chem*.**1965**, *30*, 318.
