# Supplementary material for: Di- and Tri-nuclear VIII and CrIII Complexes of Dipyridyltriazoles: Ligand Rearrangements, Mixed Valency and Ferromagnetic Coupling
Source: Front Chem. 2020 Jul 9;8:540. doi: 10.3389/fchem.2020.00540 (PMC7363982; doi:10.3389/fchem.2020.00540)

checkCIF/PLATON report

You have not supplied any structure factors. As a result the full set of tests cannot be run.

THIS REPORT IS FOR GUIDANCE ONLY. IF USED AS PART OF A REVIEW PROCEDURE FOR PUBLICATION, IT SHOULD NOT REPLACE THE EXPERTISE OF AN EXPERIENCED CRYSTALLOGRAPHIC REFEREE.

No syntax errors found. CIF dictionary Interpreting this report

Datablock: jr_33no

Bond precision: C-C = 0.0048 A Wavelength=0.71073

Cell:	a=9.8896(14)	b=10.4210(9)	c=13.397(2)
	alpha=90	beta=90	gamma=90
Temperature:	150 K		

	Calculated	Reported
Volume	1380.7(3)	1380.7(3)
Space group	P n n m	P n n m
Hall group	-P 2 2n	-P 2 2n
Moiety formula	C24 H16 Cl4 Cr2 N10	C24 H16 Cl4 Cr2 N10
Sum formula	C24 H16 Cl4 Cr2 N10	C24 H16 Cl4 Cr2 N10
Mr	690.27	690.27
Dx,g cm-3	1.660	0.000
Z	2	2
Mu (mm-1)	1.210	1.210
F000	692.0	692.0
F000’	694.59	
h,k,lmax	13,13,17	13,13,17
Nref	1730	1724
Tmin,Tmax	0.917,0.953	0.690,0.826
Tmin’	0.663	

Correction method= # Reported T Limits: Tmin=0.690 Tmax=0.826
AbsCorr = MULTI-SCAN

Data completeness= 0.997 Theta(max)= 27.957

R(reflections)= 0.0499(1408) wR2(reflections)= 0.1316(1724)

S = 1.001 Npar= 97

The following ALERTS were generated. Each ALERT has the format
test-name_ALERT_alert-type_alert-level.
Click on the hyperlinks for more details of the test.

Alert level C

```
CRYSC01_ALERT_1_C The word below has not been recognised as a standard
            identifier.
            dichroic
PLAT601_ALERT_2_C Structure Contains Solvent Accessible VOIDS of .         44 Ang**3
```

Alert level G

```
PLAT072_ALERT_2_G SHELXL First  Parameter in WGHT  Unusually Large       0.10 Report
PLAT794_ALERT_5_G Tentative Bond Valency for Cr1       (III)     .       3.09 Info
PLAT933_ALERT_2_G Number of OMIT Records in Embedded .res File ...          2 Note
```

```
   0 ALERT level A = Most likely a serious problem - resolve or explain
   0 ALERT level B = A potentially serious problem, consider carefully
   2 ALERT level C = Check. Ensure it is not caused by an omission or oversight
   3 ALERT level G = General information/check it is not something unexpected

   1 ALERT type 1 CIF construction/syntax error, inconsistent or missing data
   3 ALERT type 2 Indicator that the structure model may be wrong or deficient
   0 ALERT type 3 Indicator that the structure quality may be low
   0 ALERT type 4 Improvement, methodology, query or suggestion
   1 ALERT type 5 Informative message, check
```

It is advisable to attempt to resolve as many as possible of the alerts in all categories. Often the minor alerts point to easily fixed oversights, errors and omissions in your CIF or refinement strategy, so attention to these fine details can be worthwhile. In order to resolve some of the more serious problems it may be necessary to carry out additional measurements or structure refinements. However, the purpose of your study may justify the reported deviations and the more serious of these should normally be commented upon in the discussion or experimental section of a paper or in the "special_details" fields of the CIF. checkCIF was carefully designed to identify outliers and unusual parameters, but every test has its limitations and alerts that are not important in a particular case may appear. Conversely, the absence of alerts does not guarantee there are no aspects of the results needing attention. It is up to the individual to critically assess their own results and, if necessary, seek expert advice.

Publication of your CIF in IUCr journals

A basic structural check has been run on your CIF. These basic checks will be run on all CIFs submitted for publication in IUCr journals (*Acta Crystallographica*, *Journal of Applied Crystallography*, *Journal of Synchrotron Radiation*); however, if you intend to submit to *Acta Crystallographica Section C* or *E* or *IUCrData*, you should make sure that full publication checks are run on the final version of your CIF prior to submission.

Publication of your CIF in other journals

Please refer to the *Notes for Authors* of the relevant journal for any special instructions relating to CIF submission.

PLATON version of 23/04/2018; check.def file version of 23/04/2018

Datablock jr_33no - ellipsoid plot

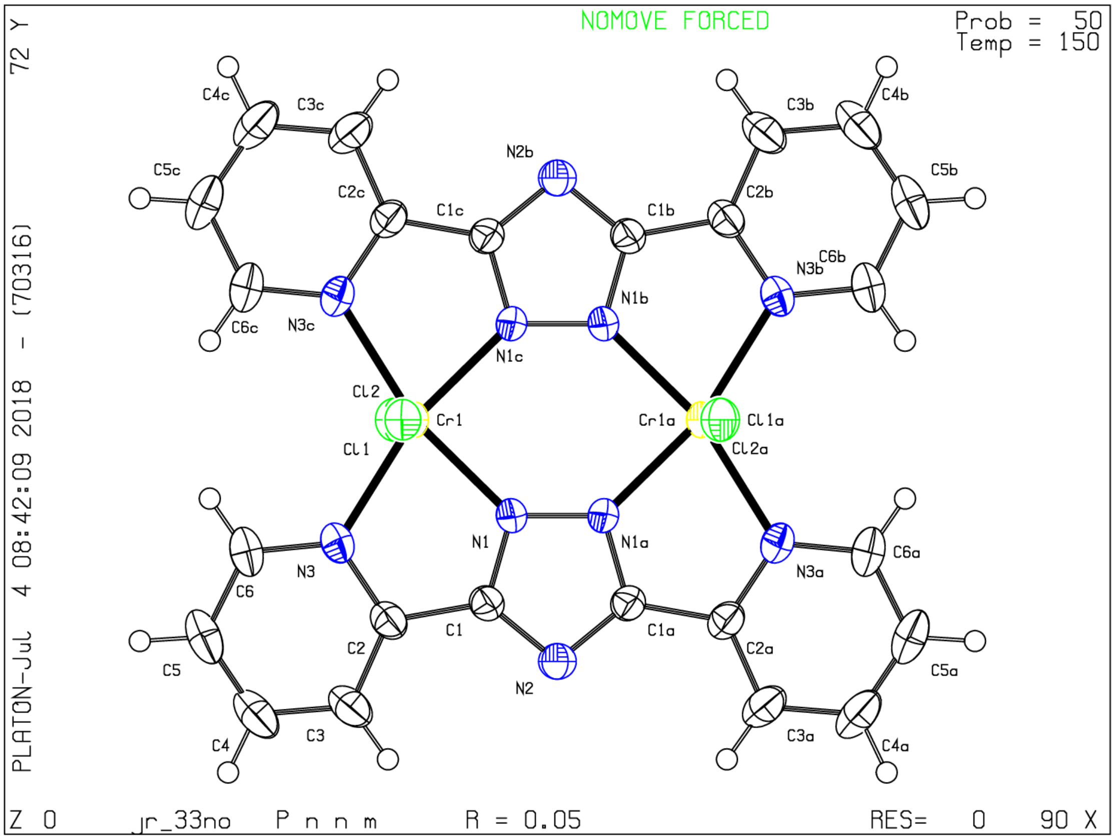

Supplement: Supplementary file 3 [file Data_Sheet_2.PDF]
