# Supplementary material for: Di- and Tri-nuclear VIII and CrIII Complexes of Dipyridyltriazoles: Ligand Rearrangements, Mixed Valency and Ferromagnetic Coupling
Source: Front Chem. 2020 Jul 9;8:540. doi: 10.3389/fchem.2020.00540 (PMC7363982; doi:10.3389/fchem.2020.00540)

checkCIF/PLATON report

You have not supplied any structure factors. As a result the full set of tests cannot be run.

THIS REPORT IS FOR GUIDANCE ONLY. IF USED AS PART OF A REVIEW PROCEDURE FOR PUBLICATION, IT SHOULD NOT REPLACE THE EXPERTISE OF AN EXPERIENCED CRYSTALLOGRAPHIC REFEREE.

No syntax errors found. CIF dictionary Interpreting this report

Datablock: jr-s4h

```
Bond precision:  C-C = 0.0058 A          Wavelength=0.71073

Cell:           a=11.3743(5)     b=38.9078(17)       c=11.0105(5)
                alpha=90         beta=102.326(1)     gamma=90
Temperature:    100 K
```

	Calculated	Reported
Volume	4760.4(4)	4760.4(4)
Space group	P 21/c	P 1 21/c 1
Hall group	-P 2ybc	-P 2ybc
Moiety formula	C36 H24 Cl6 Cr3 N15, 0.3(C H2 Cl2), 1.7(C2 H3 N)	C36 H24 Cl6 Cr3 N15, 0.3(C H2 Cl2), 1.7(C2 H3 N),
Sum formula	C39.70 H29.70 Cl6.60 Cr3 N16.70	C39.7 H29.7 Cl6.6 Cr3 N16.7
Mr	1130.67	1130.67
Dx,g cm-3	1.578	1.578
Z	4	4
Mu (mm-1)	1.094	1.094
F000	2276.0	2276.0
F000’	2284.18	
h,k,lmax	14,48,13	14,48,13
Nref	9717	9647
Tmin,Tmax	0.800,0.887	0.672,0.867
Tmin’	0.744	

```
Correction method= # Reported T Limits: Tmin=0.672 Tmax=0.867
AbsCorr = MULTI-SCAN

Data completeness= 0.993          Theta(max)= 26.347

R(reflections)= 0.0548( 9052)     wR2(reflections)= 0.1172( 9647)

S = 1.309                  Npar= 619
```

```
The following ALERTS were generated. Each ALERT has the format
       test-name_ALERT_alert-type_alert-level.
Click on the hyperlinks for more details of the test.
```

Alert level C

```
PLAT041_ALERT_1_C Calc. and Reported SumFormula    Strings  Differ     Please Check
PLAT077_ALERT_4_C Unitcell Contains Non-integer Number of Atoms ..     Please Check
```

Alert level G

```
PLAT002_ALERT_2_G Number of Distance or Angle Restraints on AtSite          9 Note
PLAT003_ALERT_2_G Number of Uiso or Uij Restrained non-H Atoms ...          6 Report
PLAT083_ALERT_2_G SHELXL Second Parameter in WGHT  Unusually Large      14.32 Why ?
PLAT175_ALERT_4_G The CIF-Embedded .res File Contains SAME Records          1 Report
PLAT176_ALERT_4_G The CIF-Embedded .res File Contains SADI Records          1 Report
PLAT178_ALERT_4_G The CIF-Embedded .res File Contains SIMU Records          1 Report
PLAT187_ALERT_4_G The CIF-Embedded .res File Contains RIGU Records          2 Report
PLAT300_ALERT_4_G Atom Site Occupancy of Cl7B       Constrained at        0.3 Check
PLAT300_ALERT_4_G Atom Site Occupancy of Cl8B       Constrained at        0.3 Check
PLAT300_ALERT_4_G Atom Site Occupancy of C51B       Constrained at        0.3 Check
PLAT300_ALERT_4_G Atom Site Occupancy of H51A       Constrained at        0.3 Check
PLAT300_ALERT_4_G Atom Site Occupancy of H51B       Constrained at        0.3 Check
PLAT300_ALERT_4_G Atom Site Occupancy of N51A       Constrained at        0.7 Check
PLAT300_ALERT_4_G Atom Site Occupancy of C51A       Constrained at        0.7 Check
PLAT300_ALERT_4_G Atom Site Occupancy of C52A       Constrained at        0.7 Check
PLAT300_ALERT_4_G Atom Site Occupancy of H52A       Constrained at        0.7 Check
PLAT300_ALERT_4_G Atom Site Occupancy of H52B       Constrained at        0.7 Check
PLAT300_ALERT_4_G Atom Site Occupancy of H52C       Constrained at        0.7 Check
PLAT302_ALERT_4_G Anion/Solvent/Minor-Residue Disorder (Resd  2  )       100% Note
PLAT302_ALERT_4_G Anion/Solvent/Minor-Residue Disorder (Resd  4  )       100% Note
PLAT304_ALERT_4_G Non-Integer Number of Atoms in ...... Resd  2          1.50 Check
PLAT304_ALERT_4_G Non-Integer Number of Atoms in ...... Resd  4          4.20 Check
PLAT432_ALERT_2_G Short Inter X...Y Contact  C24      ..C24              3.18 Ang.
PLAT794_ALERT_5_G Tentative Bond Valency for Cr1       (III)     .       3.08 Info
PLAT794_ALERT_5_G Tentative Bond Valency for Cr2       (III)     .       3.12 Info
PLAT794_ALERT_5_G Tentative Bond Valency for Cr3       (III)     .       3.07 Info
PLAT860_ALERT_3_G Number of Least-Squares Restraints .............         19 Note
PLAT933_ALERT_2_G Number of OMIT Records in Embedded .res File ...          8 Note
```

---

0 **ALERT level A** = Most likely a serious problem - resolve or explain
0 **ALERT level B** = A potentially serious problem, consider carefully
2 **ALERT level C** = Check. Ensure it is not caused by an omission or oversight
28 **ALERT level G** = General information/check it is not something unexpected

1 ALERT type 1 CIF construction/syntax error, inconsistent or missing data
5 ALERT type 2 Indicator that the structure model may be wrong or deficient
1 ALERT type 3 Indicator that the structure quality may be low
20 ALERT type 4 Improvement, methodology, query or suggestion
3 ALERT type 5 Informative message, check

It is advisable to attempt to resolve as many as possible of the alerts in all categories. Often the minor alerts point to easily fixed oversights, errors and omissions in your CIF or refinement strategy, so attention to these fine details can be worthwhile. In order to resolve some of the more serious problems it may be necessary to carry out additional measurements or structure refinements. However, the purpose of your study may justify the reported deviations and the more serious of these should normally be commented upon in the discussion or experimental section of a paper or in the "special_details" fields of the CIF. checkCIF was carefully designed to identify outliers and unusual parameters, but every test has its limitations and alerts that are not important in a particular case may appear. Conversely, the absence of alerts does not guarantee there are no aspects of the results needing attention. It is up to the individual to critically assess their own results and, if necessary, seek expert advice.

Publication of your CIF in IUCr journals

A basic structural check has been run on your CIF. These basic checks will be run on all CIFs submitted for publication in IUCr journals (*Acta Crystallographica*, *Journal of Applied Crystallography*, *Journal of Synchrotron Radiation*); however, if you intend to submit to *Acta Crystallographica Section C* or *E* or *IUCrData*, you should make sure that full publication checks are run on the final version of your CIF prior to submission.

Publication of your CIF in other journals

Please refer to the *Notes for Authors* of the relevant journal for any special instructions relating to CIF submission.

PLATON version of 23/04/2018; check.def file version of 23/04/2018

Datablock jr-s4h - ellipsoid plot

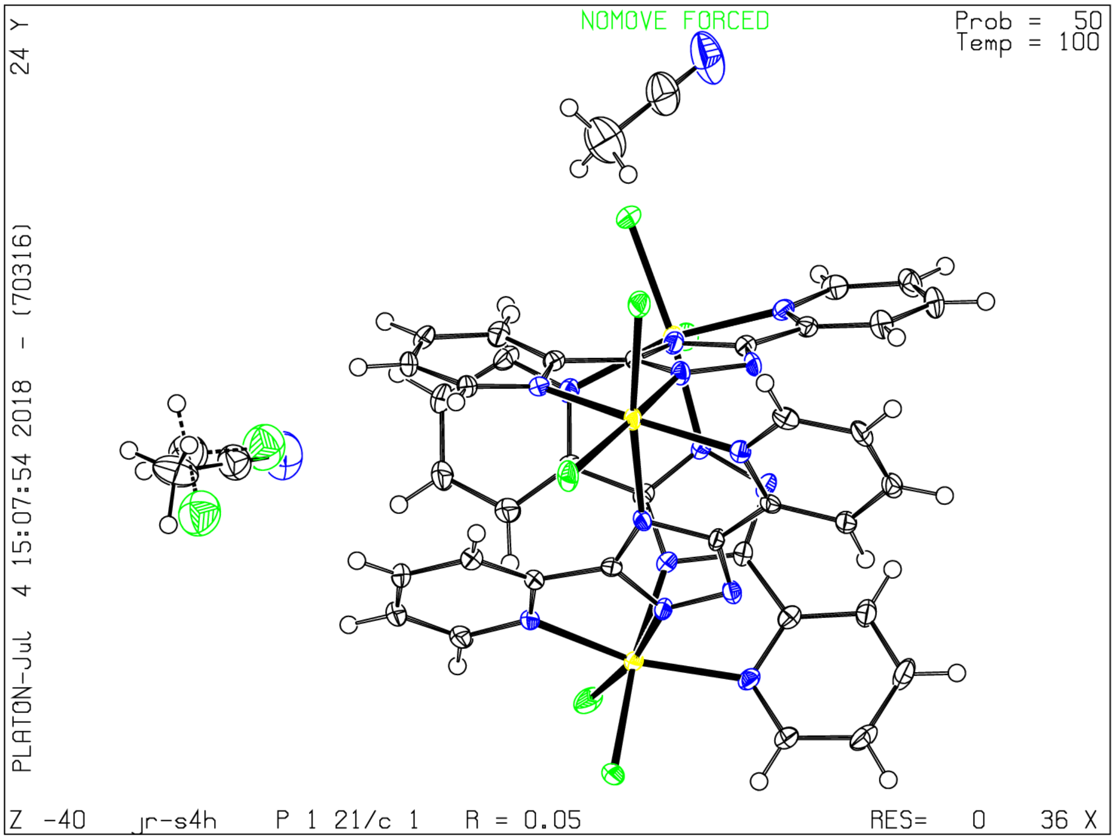

Supplement: Supplementary file 4 [file Data_Sheet_3.PDF]
