# Supplementary material for: Di- and Tri-nuclear VIII and CrIII Complexes of Dipyridyltriazoles: Ligand Rearrangements, Mixed Valency and Ferromagnetic Coupling
Source: Front Chem. 2020 Jul 9;8:540. doi: 10.3389/fchem.2020.00540 (PMC7363982; doi:10.3389/fchem.2020.00540)

checkCIF/PLATON report

You have not supplied any structure factors. As a result the full set of tests cannot be run.

THIS REPORT IS FOR GUIDANCE ONLY. IF USED AS PART OF A REVIEW PROCEDURE FOR PUBLICATION, IT SHOULD NOT REPLACE THE EXPERTISE OF AN EXPERIENCED CRYSTALLOGRAPHIC REFEREE.

No syntax errors found. CIF dictionary Interpreting this report

Datablock: jrs146

```
Bond precision:  C-C = 0.0050 A          Wavelength=0.71073

Cell:           a=8.853(2)          b=11.014(3)       c=12.255(3)
                alpha=69.267(18)    beta=70.330(19)   gamma=73.229(19)
Temperature:    150 K
```

	Calculated	Reported
Volume	1032.7(5)	1032.7(5)
Space group	P -1	P -1
Hall group	-P 1	-P 1
Moiety formula	C34 H24 Cl6 N12 V2, 0.2(C H2 Cl2), 1.8(C2 H3 N)	C34 H24 Cl6 N12 V2, 1.8(C2 H3 N), 0.2(C H2 Cl2)
Sum formula	C37.80 H29.80 Cl6.40 N13.80 V2	C37.80 H29.80 Cl6.40 N13.80 V2
Mr	1006.11	1006.11
Dx,g cm-3	1.618	1.618
Z	1	1
Mu (mm-1)	0.916	0.916
F000	508.0	508.0
F000’	509.65	
h,k,lmax	11,13,15	11,13,15
Nref	4425	4362
Tmin,Tmax	0.839,0.896	0.687,0.900
Tmin’	0.713	

```
Correction method= # Reported T Limits: Tmin=0.687 Tmax=0.900
AbsCorr = MULTI-SCAN

Data completeness= 0.986          Theta(max)= 26.823

R(reflections)= 0.0574( 3401)     wR2(reflections)= 0.1501( 4362)

S = 0.989                   Npar= 284
```

```
The following ALERTS were generated. Each ALERT has the format
       test-name_ALERT_alert-type_alert-level.
Click on the hyperlinks for more details of the test.
```

Alert level C

```
PLAT077_ALERT_4_C Unitcell Contains Non-integer Number of Atoms ..     Please Check
```

Alert level G

```
PLAT002_ALERT_2_G Number of Distance or Angle Restraints on AtSite          3 Note
PLAT003_ALERT_2_G Number of Uiso or Uij Restrained non-H Atoms ...          4 Report
PLAT042_ALERT_1_G Calc. and Reported MoietyFormula Strings  Differ     Please Check
PLAT072_ALERT_2_G SHELXL First  Parameter in WGHT  Unusually Large       0.11 Report
PLAT172_ALERT_4_G The CIF-Embedded .res File Contains DFIX Records          1 Report
PLAT178_ALERT_4_G The CIF-Embedded .res File Contains SIMU Records          1 Report
PLAT187_ALERT_4_G The CIF-Embedded .res File Contains RIGU Records          1 Report
PLAT300_ALERT_4_G Atom Site Occupancy of Cl4B       Constrained at        0.1 Check
PLAT300_ALERT_4_G Atom Site Occupancy of Cl5B       Constrained at        0.1 Check
PLAT300_ALERT_4_G Atom Site Occupancy of C21B       Constrained at        0.1 Check
PLAT300_ALERT_4_G Atom Site Occupancy of H21A       Constrained at        0.1 Check
PLAT300_ALERT_4_G Atom Site Occupancy of H21B       Constrained at        0.1 Check
PLAT300_ALERT_4_G Atom Site Occupancy of N21A       Constrained at        0.9 Check
PLAT300_ALERT_4_G Atom Site Occupancy of C21A       Constrained at        0.9 Check
PLAT300_ALERT_4_G Atom Site Occupancy of C22A       Constrained at        0.9 Check
PLAT300_ALERT_4_G Atom Site Occupancy of H22A       Constrained at        0.9 Check
PLAT300_ALERT_4_G Atom Site Occupancy of H22B       Constrained at        0.9 Check
PLAT300_ALERT_4_G Atom Site Occupancy of H22C       Constrained at        0.9 Check
PLAT302_ALERT_4_G Anion/Solvent/Minor-Residue Disorder (Resd  2  )       100% Note
PLAT302_ALERT_4_G Anion/Solvent/Minor-Residue Disorder (Resd  3  )       100% Note
PLAT304_ALERT_4_G Non-Integer Number of Atoms in ...... Resd  2          0.50 Check
PLAT304_ALERT_4_G Non-Integer Number of Atoms in ...... Resd  3          5.40 Check
PLAT794_ALERT_5_G Tentative Bond Valency for V1        (III)     .       3.30 Info
PLAT860_ALERT_3_G Number of Least-Squares Restraints .............         17 Note
PLAT933_ALERT_2_G Number of OMIT Records in Embedded .res File ...          4 Note
```

```
   0 ALERT level A = Most likely a serious problem - resolve or explain
   0 ALERT level B = A potentially serious problem, consider carefully
   1 ALERT level C = Check. Ensure it is not caused by an omission or oversight
  25 ALERT level G = General information/check it is not something unexpected

   1 ALERT type 1 CIF construction/syntax error, inconsistent or missing data
   4 ALERT type 2 Indicator that the structure model may be wrong or deficient
   1 ALERT type 3 Indicator that the structure quality may be low
  19 ALERT type 4 Improvement, methodology, query or suggestion
   1 ALERT type 5 Informative message, check
```

It is advisable to attempt to resolve as many as possible of the alerts in all categories. Often the minor alerts point to easily fixed oversights, errors and omissions in your CIF or refinement strategy, so attention to these fine details can be worthwhile. In order to resolve some of the more serious problems it may be necessary to carry out additional measurements or structure refinements. However, the purpose of your study may justify the reported deviations and the more serious of these should normally be commented upon in the discussion or experimental section of a paper or in the "special_details" fields of the CIF. checkCIF was carefully designed to identify outliers and unusual parameters, but every test has its limitations and alerts that are not important in a particular case may appear. Conversely, the absence of alerts does not guarantee there are no aspects of the results needing attention. It is up to the individual to critically assess their own results and, if necessary, seek expert advice.

Publication of your CIF in IUCr journals

A basic structural check has been run on your CIF. These basic checks will be run on all CIFs submitted for publication in IUCr journals (*Acta Crystallographica*, *Journal of Applied Crystallography*, *Journal of Synchrotron Radiation*); however, if you intend to submit to *Acta Crystallographica Section C* or *E* or *IUCrData*, you should make sure that full publication checks are run on the final version of your CIF prior to submission.

Publication of your CIF in other journals

Please refer to the *Notes for Authors* of the relevant journal for any special instructions relating to CIF submission.

PLATON version of 23/04/2018; check.def file version of 23/04/2018

Datablock jrs146 - ellipsoid plot

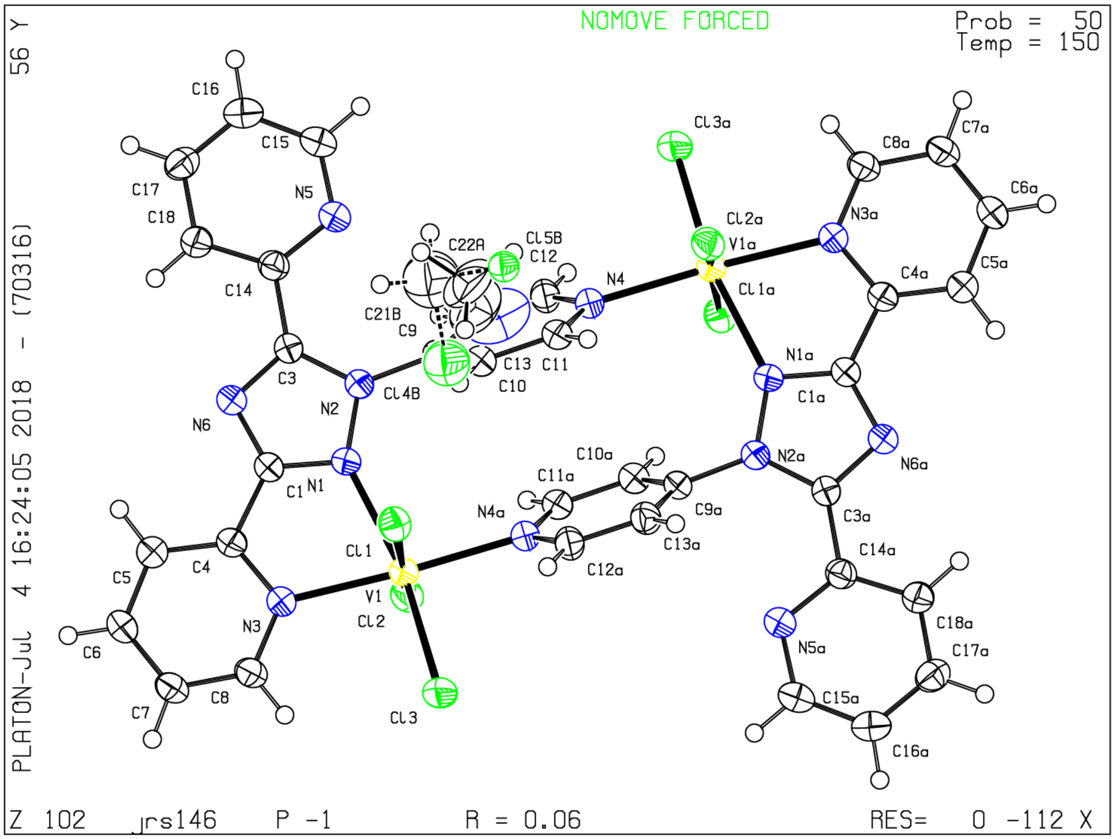

Supplement: Supplementary file 5 [file Data_Sheet_4.PDF]
