# Supplementary material for: Di- and Tri-nuclear VIII and CrIII Complexes of Dipyridyltriazoles: Ligand Rearrangements, Mixed Valency and Ferromagnetic Coupling
Source: Front Chem. 2020 Jul 9;8:540. doi: 10.3389/fchem.2020.00540 (PMC7363982; doi:10.3389/fchem.2020.00540)

checkCIF/PLATON report

You have not supplied any structure factors. As a result the full set of tests cannot be run.

THIS REPORT IS FOR GUIDANCE ONLY. IF USED AS PART OF A REVIEW PROCEDURE FOR PUBLICATION, IT SHOULD NOT REPLACE THE EXPERTISE OF AN EXPERIENCED CRYSTALLOGRAPHIC REFEREE.

No syntax errors found.   CIF dictionary   Interpreting this report

Datablock: jrs66ox

```
Bond precision:  C-C = 0.0047 A         Wavelength=0.71073

Cell:          a=8.8302(9)       b=11.0282(11)    c=12.2533(12)
               alpha=67.901(2)   beta=69.489(2)   gamma=73.071(2)
Temperature:   100 K
```

	Calculated	Reported
Volume	1017.88(18)	1017.88(18)
Space group	P -1	P -1
Hall group	-P 1	-P 1
Moiety formula	C34 H24 Cl5.16 N12 O0.84 V2, 1.16(C2 H3 N), 0.84(O)	C34 H24 Cl5.16 N12 O0.84 V2, 1.16(C2 H3 N), 0.84(H2 O)
Sum formula	C36.32 H27.48 Cl5.16 N13.16 O1.68 V2	C36.32 H29.16 Cl5.16 N13.16 O1.68 V2
Mr	959.96	961.65
Dx,g cm-3	1.566	1.569
Z	1	1
Mu (mm-1)	0.849	0.849
F000	484.7	486.0
F000’	486.16	
h,k,lmax	11,14,16	11,14,16
Nref	4904	4349
Tmin,Tmax	0.894,0.926	0.787,0.928
Tmin’	0.851	

```
Correction method= # Reported T Limits: Tmin=0.787 Tmax=0.928
AbsCorr = MULTI-SCAN

Data completeness= 0.887          Theta(max)= 27.972

R(reflections)= 0.0521( 4032)     wR2(reflections)= 0.1278( 4349)

S = 1.135                   Npar= 270
```

The following ALERTS were generated. Each ALERT has the format
test-name_ALERT_alert-type_alert-level.
Click on the hyperlinks for more details of the test.

Alert level C

PLAT041_ALERT_1_C Calc. and Reported SumFormula Strings Differ Please Check
PLAT043_ALERT_1_C Calculated and Reported Mol. Weight Differ by .. 1.69 Check
PLAT077_ALERT_4_C Unitcell Contains Non-integer Number of Atoms .. Please Check
PLAT202_ALERT_3_C Isotropic non-H Atoms in Anion/Solvent .... 3 Check

Alert level G

FORMU01_ALERT_2_G There is a discrepancy between the atom counts in the
_chemical_formula_sum and the formula from the _atom_site* data.
Atom count from _chemical_formula_sum:C36.32 H29.16 Cl5.16 N13.16 O1
Atom count from the _atom_site data: C36.32 H27.48 Cl5.16 N13.16 O1
CELLZ01_ALERT_1_G Difference between formula and atom_site contents detected.
CELLZ01_ALERT_1_G WARNING: H atoms missing from atom site list. Is this intentional?
From the CIF: _cell_formula_units_Z 1
From the CIF: _chemical_formula_sum C36.32 H29.16 Cl5.16 N13.16 O1.6
TEST: Compare cell contents of formula and atom_site data

atom	Z*formula	cif sites	diff
C	36.32	36.32	0.00
H	29.16	27.48	1.68
Cl	5.16	5.16	0.00
N	13.16	13.16	0.00
O	1.68	1.68	0.00
V	2.00	2.00	0.00

PLAT002_ALERT_2_G Number of Distance or Angle Restraints on AtSite 5 Note
PLAT003_ALERT_2_G Number of Uiso or Uij Restrained non-H Atoms ... 3 Report
PLAT042_ALERT_1_G Calc. and Reported MoietyFormula Strings Differ Please Check
PLAT068_ALERT_1_G Reported F000 Differs from Calcd (or Missing)... Please Check
PLAT154_ALERT_1_G The s.u.’s on the Cell Angles are Equal ..(Note) 0.002 Degree
PLAT172_ALERT_4_G The CIF-Embedded .res File Contains DFIX Records 3 Report
PLAT177_ALERT_4_G The CIF-Embedded .res File Contains DELU Records 1 Report
PLAT232_ALERT_2_G Hirshfeld Test Diff (M-X) V1 --Cl3A . 18.1 s.u.
PLAT232_ALERT_2_G Hirshfeld Test Diff (M-X) V1 --N1 . 12.8 s.u.
PLAT300_ALERT_4_G Atom Site Occupancy of Cl3A Constrained at 0.58 Check
PLAT300_ALERT_4_G Atom Site Occupancy of O3B Constrained at 0.42 Check
PLAT300_ALERT_4_G Atom Site Occupancy of N21A Constrained at 0.58 Check
PLAT300_ALERT_4_G Atom Site Occupancy of C21A Constrained at 0.58 Check
PLAT300_ALERT_4_G Atom Site Occupancy of C22A Constrained at 0.58 Check
PLAT300_ALERT_4_G Atom Site Occupancy of H22A Constrained at 0.58 Check
PLAT300_ALERT_4_G Atom Site Occupancy of H22B Constrained at 0.58 Check
PLAT300_ALERT_4_G Atom Site Occupancy of H22C Constrained at 0.58 Check
PLAT300_ALERT_4_G Atom Site Occupancy of O21B Constrained at 0.42 Check
PLAT301_ALERT_3_G Main Residue Disorder ....(Resd 1) 4% Note
PLAT302_ALERT_4_G Anion/Solvent/Minor-Residue Disorder (Resd 2) 100% Note
PLAT302_ALERT_4_G Anion/Solvent/Minor-Residue Disorder (Resd 3) 100% Note
PLAT304_ALERT_4_G Non-Integer Number of Atoms in ...... Resd 2 3.48 Check
PLAT304_ALERT_4_G Non-Integer Number of Atoms in ...... Resd 3 0.42 Check
PLAT311_ALERT_2_G Isolated Disordered Oxygen Atom (No H’s ?) ..... O21B Check
PLAT790_ALERT_4_G Centre of Gravity not Within Unit Cell: Resd. # 2 Note
C2 H3 N
PLAT790_ALERT_4_G Centre of Gravity not Within Unit Cell: Resd. # 3 Note
O
PLAT860_ALERT_3_G Number of Least-Squares Restraints .... 5 Note
PLAT933_ALERT_2_G Number of OMIT Records in Embedded .res File ... 2 Note

```
 0 ALERT level A = Most likely a serious problem - resolve or explain
 0 ALERT level B = A potentially serious problem, consider carefully
 4 ALERT level C = Check. Ensure it is not caused by an omission or oversight
31 ALERT level G = General information/check it is not something unexpected

 7 ALERT type 1 CIF construction/syntax error, inconsistent or missing data
 7 ALERT type 2 Indicator that the structure model may be wrong or deficient
 3 ALERT type 3 Indicator that the structure quality may be low
18 ALERT type 4 Improvement, methodology, query or suggestion
 0 ALERT type 5 Informative message, check
```

It is advisable to attempt to resolve as many as possible of the alerts in all categories. Often the minor alerts point to easily fixed oversights, errors and omissions in your CIF or refinement strategy, so attention to these fine details can be worthwhile. In order to resolve some of the more serious problems it may be necessary to carry out additional measurements or structure refinements. However, the purpose of your study may justify the reported deviations and the more serious of these should normally be commented upon in the discussion or experimental section of a paper or in the "special_details" fields of the CIF. checkCIF was carefully designed to identify outliers and unusual parameters, but every test has its limitations and alerts that are not important in a particular case may appear. Conversely, the absence of alerts does not guarantee there are no aspects of the results needing attention. It is up to the individual to critically assess their own results and, if necessary, seek expert advice.

Publication of your CIF in IUCr journals

A basic structural check has been run on your CIF. These basic checks will be run on all CIFs submitted for publication in IUCr journals (*Acta Crystallographica*, *Journal of Applied Crystallography*, *Journal of Synchrotron Radiation*); however, if you intend to submit to *Acta Crystallographica Section C* or *E* or *IUCrData*, you should make sure that full publication checks are run on the final version of your CIF prior to submission.

Publication of your CIF in other journals

Please refer to the *Notes for Authors* of the relevant journal for any special instructions relating to CIF submission.

PLATON version of 23/04/2018; check.def file version of 23/04/2018

Datablock jrs66ox - ellipsoid plot

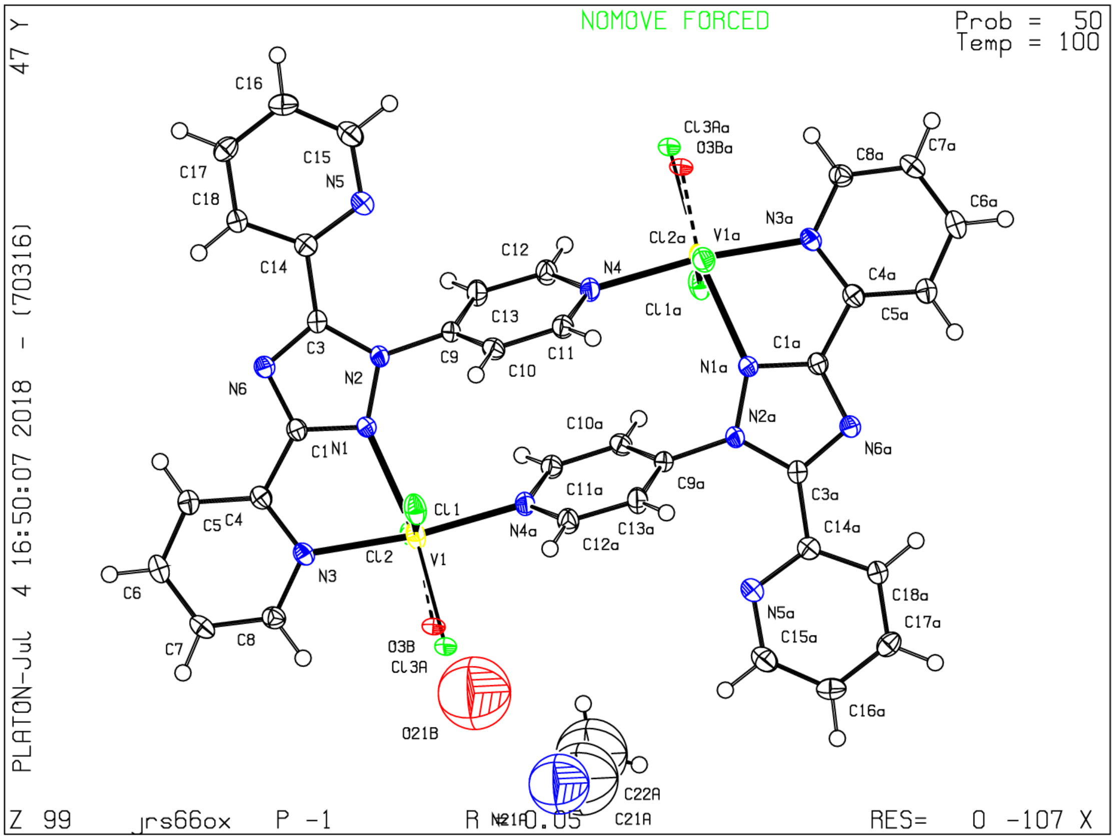

Supplement: Supplementary file 6 [file Data_Sheet_5.PDF]
