# Supplementary material for: Di- and Tri-nuclear VIII and CrIII Complexes of Dipyridyltriazoles: Ligand Rearrangements, Mixed Valency and Ferromagnetic Coupling
Source: Front Chem. 2020 Jul 9;8:540. doi: 10.3389/fchem.2020.00540 (PMC7363982; doi:10.3389/fchem.2020.00540)

checkCIF/PLATON report

You have not supplied any structure factors. As a result the full set of tests cannot be run.

THIS REPORT IS FOR GUIDANCE ONLY. IF USED AS PART OF A REVIEW PROCEDURE FOR PUBLICATION, IT SHOULD NOT REPLACE THE EXPERTISE OF AN EXPERIENCED CRYSTALLOGRAPHIC REFEREE.

No syntax errors found. CIF dictionary Interpreting this report

Datablock: abc233

```
Bond precision:  C-C = 0.0041 A          Wavelength=1.54184

Cell:            a=12.5048(3)     b=14.6842(3)     c=20.7147(5)
                 alpha=90         beta=90          gamma=90
Temperature:     200 K
```

	Calculated	Reported
Volume	3803.70(15)	3803.70(15)
Space group	P b c a	P b c a
Hall group	-P 2ac 2ab	-P 2ac 2ab
Moiety formula	C34 H24 Cl5.64 N12 O0.36 V2	C34 H24 Cl5.64 N12 O0.36 V2
Sum formula	C34 H24 Cl5.64 N12 O0.36 V2	C34 H24 Cl5.64 N12 O0.36 V2
Mr	908.23	908.23
Dx,g cm-3	1.586	1.586
Z	4	4
Mu (mm-1)	8.145	8.145
F000	1827.0	1827.0
F000’	1839.76	
h,k,lmax	15,18,25	15,18,25
Nref	3678	3606
Tmin,Tmax	0.237,0.347	0.307,1.000
Tmin’	0.043	

```
Correction method= # Reported T Limits: Tmin=0.307 Tmax=1.000
AbsCorr = MULTI-SCAN

Data completeness= 0.980          Theta(max)= 71.074

R(reflections)= 0.0398( 3234)     wR2(reflections)= 0.1063( 3606)

S = 1.040                   Npar= 297
```

The following ALERTS were generated. Each ALERT has the format
test-name_ALERT_alert-type_alert-level.
Click on the hyperlinks for more details of the test.

Alert level C

```
PLAT077_ALERT_4_C Unitcell Contains Non-integer Number of Atoms ..     Please Check
```

Alert level G

```
PLAT003_ALERT_2_G Number of Uiso or Uij Restrained non-H Atoms ...          3 Report
PLAT164_ALERT_4_G Nr. of Refined C-H H-Atoms in Heavy-Atom Struct.         12 Note
PLAT178_ALERT_4_G The CIF-Embedded .res File Contains SIMU Records          1 Report
PLAT187_ALERT_4_G The CIF-Embedded .res File Contains RIGU Records          1 Report
PLAT232_ALERT_2_G Hirshfeld Test Diff (M-X)  V1       --Cl3A     .       18.0 s.u.
PLAT232_ALERT_2_G Hirshfeld Test Diff (M-X)  V1       --N1       .        9.2 s.u.
PLAT300_ALERT_4_G Atom Site Occupancy of Cl3A       Constrained at       0.82 Check
PLAT300_ALERT_4_G Atom Site Occupancy of O3B        Constrained at       0.18 Check
PLAT301_ALERT_3_G Main Residue  Disorder ..............(Resd  1  )         4% Note
PLAT860_ALERT_3_G Number of Least-Squares Restraints .............          9 Note
```

---

0 **ALERT level A** = Most likely a serious problem - resolve or explain
0 **ALERT level B** = A potentially serious problem, consider carefully
1 **ALERT level C** = Check. Ensure it is not caused by an omission or oversight
10 **ALERT level G** = General information/check it is not something unexpected

0 ALERT type 1 CIF construction/syntax error, inconsistent or missing data
3 ALERT type 2 Indicator that the structure model may be wrong or deficient
2 ALERT type 3 Indicator that the structure quality may be low
6 ALERT type 4 Improvement, methodology, query or suggestion
0 ALERT type 5 Informative message, check

It is advisable to attempt to resolve as many as possible of the alerts in all categories. Often the minor alerts point to easily fixed oversights, errors and omissions in your CIF or refinement strategy, so attention to these fine details can be worthwhile. In order to resolve some of the more serious problems it may be necessary to carry out additional measurements or structure refinements. However, the purpose of your study may justify the reported deviations and the more serious of these should normally be commented upon in the discussion or experimental section of a paper or in the "special_details" fields of the CIF. checkCIF was carefully designed to identify outliers and unusual parameters, but every test has its limitations and alerts that are not important in a particular case may appear. Conversely, the absence of alerts does not guarantee there are no aspects of the results needing attention. It is up to the individual to critically assess their own results and, if necessary, seek expert advice.

Publication of your CIF in IUCr journals

A basic structural check has been run on your CIF. These basic checks will be run on all CIFs submitted for publication in IUCr journals (*Acta Crystallographica*, *Journal of Applied Crystallography*, *Journal of Synchrotron Radiation*); however, if you intend to submit to *Acta Crystallographica Section C* or *E* or *IUCrData*, you should make sure that full publication checks are run on the final version of your CIF prior to submission.

Publication of your CIF in other journals

Please refer to the *Notes for Authors* of the relevant journal for any special instructions relating to CIF submission.

PLATON version of 22/12/2019; check.def file version of 13/12/2019

Datablock abc233 - ellipsoid plot

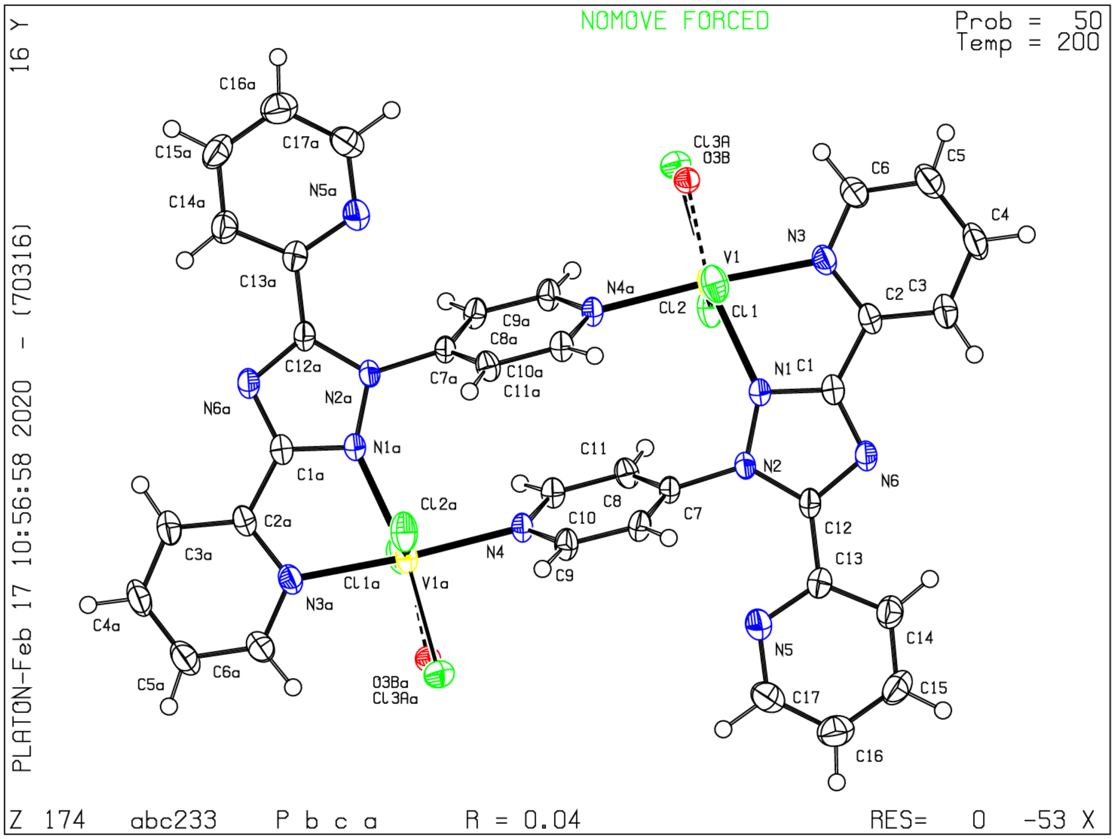

Supplement: Supplementary file 7 [file Data_Sheet_6.PDF]
